# Design and Evaluation of Meningococcal Vaccines through Structure-Based Modification of Host and Pathogen Molecules

**DOI:** 10.1371/journal.ppat.1002981

**Published:** 2012-10-25

**Authors:** Steven Johnson, Lionel Tan, Stijn van der Veen, Joseph Caesar, Elena Goicoechea De Jorge, Rachel J. Harding, Xilian Bai, Rachel M. Exley, Philip N. Ward, Nicola Ruivo, Kaushali Trivedi, Elspeth Cumber, Rhian Jones, Luke Newham, David Staunton, Rafael Ufret-Vincenty, Ray Borrow, Matthew C. Pickering, Susan M. Lea, Christoph M. Tang

**Affiliations:** 1 Sir William Dunn School of Pathology, University of Oxford, Oxford, United Kingdom; 2 Centre for Molecular Microbiology and Infection, Imperial College, London, United Kingdom; 3 Centre for Complement and Inflammation Research (CCIR), Department of Medicine, Imperial College, London, United Kingdom; 4 Vaccine Evaluation Unit, Public Health Laboratory, Manchester Medical Microbiology Partnership, Manchester, United Kingdom; 5 Department of Biochemistry, University of Oxford, Oxford, United Kingdom; 6 Department of Ophthalmology, UT Southwestern Medical Center, Dallas, Texas, United States of America; Faculté de Médecine Paris Descartes, site Necker, France

## Abstract

*Neisseria meningitis* remains a leading cause of sepsis and meningitis, and vaccines are required to prevent infections by this important human pathogen. Factor H binding protein (fHbp) is a key antigen that elicits protective immunity against the meningococcus and recruits the host complement regulator, fH. As the high affinity interaction between fHbp and fH could impair immune responses, we sought to identify non-functional fHbps that could act as effective immunogens. This was achieved by alanine substitution of fHbps from all three variant groups (V1, V2 and V3 fHbp) of the protein; while some residues affected fH binding in each variant group, the distribution of key amino underlying the interaction with fH differed between the V1, V2 and V3 proteins. The atomic structure of V3 fHbp in complex with fH and of the C-terminal barrel of V2 fHbp provide explanations to the differences in the precise nature of their interactions with fH, and the instability of the V2 protein. To develop transgenic models to assess the efficacy of non-functional fHbps, we determined the structural basis of the low level of interaction between fHbp and murine fH; in addition to changes in amino acids in the fHbp binding site, murine fH has a distinct conformation compared with the human protein that would sterically inhibit binding to fHbp. Non-functional V1 fHbps were further characterised by binding and structural studies, and shown in non-transgenic and transgenic mice (expressing chimeric fH that binds fHbp and precisely regulates complement system) to retain their immunogenicity. Our findings provide a catalogue of non-functional fHbps from all variant groups that can be included in new generation meningococcal vaccines, and establish proof-in-principle for clinical studies to compare their efficacy with wild-type fHbps.

## Author Summary

Vaccines are currently available against several serogroups of *Neisseria meningitidis*. However broadly effective serogroup B vaccines are still required as capsule-based approaches cannot be implemented with this serogroup because of the risks of auto-immunity. As a result, vaccines based on proteins in the bacterial outer membrane are being developed. Factor H binding protein (fHbp) is an important meningococcal immunogen which is able to bind the human complement regulator factor H (fH) at high affinity; this interaction could impair the efficacy of fHbp-based vaccines. Here we perform structure:function analyses to define non-functional fHbps and to explain the basis for the host specificity of the fHbp:fH interaction. The vaccine candidacy of non-functional fHbps was compared with wild-type proteins in a relevant transgenic model. These findings should allow the design and evaluation of future fHbp vaccines against this important human pathogen.

## Introduction

*Neisseria meningitidis* is a human specific pathogen that is a leading cause of bacteraemia and sepsis in children and young adults [1]. The initial symptoms of meningococcal disease are non-specific, so the diagnosis is often missed in its early stages; infection can then progress rapidly over only a few hours in severe cases [2]. Mortality rates remain high despite optimal medical therapy, with septicaemia associated with a 10% case fatality [3]. These features mean that prophylactic vaccination remains the best approach to protect individuals from this important human pathogen [4].

Considerable progress has been made in the development of conjugate capsular polysaccharide vaccines against certain serogroups of *N. meningitidis* (namely A, C, Y and W135), while outer membrane vesicle (OMV) vaccines have been successfully employed to combat epidemic disease caused by a single clones of the bacterium [5]. However, these strategies cannot be employed to prevent endemic serogroup B infection, which is the commonest form of disease in countries across Europe and North America [1], [6]. This is because of the structural identity of the α2–8 linked polysialic acid serogroup B capsule with a modification on human N-CAM1, preventing its use as an immunogen because of fears of autoimmunity [7]. Furthermore the phenotypic diversity of serogroup B strains limits the potential efficacy of OMV vaccines [5].

As a consequence, there have been considerable efforts to identify sub-capsular antigens as vaccine candidates that elicit appropriate immune responses. Pioneering studies with serogroup C strains have demonstrated that the serum bactericidal antibodies (SBA), that develop either naturally (following carriage of bacteria) or through immunisation, are sufficient to provide protection against meningococcal disease [8], [9].

Factor H binding protein (fHbp) is a 27 kDa surface lipoprotein consisting of two *β*-barrels [10] that promotes resistance against complement mediated lysis [11] and is a key meningococcal antigen that elicits SBAs [12], [13]. It is a component of two serogroup B vaccines undergoing Phase III clinical trials; one vaccine contains fHbp alone, while the other consists of a single fHbp in combination with other protein antigens as well as an OMV [5]. fHbp can be divided into three variant groups, V1, V2, and V3 [13], or two sub-families [14] based on its predicted amino acid sequence. fHbps belonging to the same variant group share over 85% amino acid identity, and only 60–70% similarity between variant groups. Furthermore immunisation with a protein belonging to one variant family generates responses with some immunological cross-reactivity within, but not between, variant groups [12], [13].

fHbp binds the complement regulatory molecule factor H (fH) at high affinity, with a dissociation constant (*K*_D_) in the nanomolar range [10], tighter than for any other known fH ligand. fH is the major regulator of the alternative pathway (AP) of complement activation; this pathway is critical to complement homeostasis as it serves to amplify activation initiated by the recognition of foreign antigens by antibodies or lectins [15]. fH consists of 20 complement control protein domains (CCP), each of approximately 60 amino acids, and joined by short linker sequences. Different CCPs possess distinct functions and interact with cognate partners [16], precisely modulating their activity to mediate the diverse regulatory roles of fH (as a co-factor for fI mediated cleavage of C3b and a decay accelerating factor) [16], [17]. Although structure:function studies have been performed to characterise V1 proteins and their interaction with fH [10], [18], little is known about V2 and V3 fHbps. We have shown previously that fH CCP 6 and 7 (fH_67_) are necessary for high affinity interactions with V1 fHbp, and that these two CCPs are sufficient to inhibit binding of full length fH to fHbp [10]. As fHbp binds human (hfH) but not murine fH (mfH), novel models are required to assess the efficacy of fHbp-based vaccines. However, it is not sufficient to simply introduce a gene encoding hfH into rodents, as it is not known how this molecule will bind and regulate murine complement factors [19].

Binding of fH to fHbp could affect its efficacy as a vaccine given the high affinity of the interaction, serum levels of fH (the second most abundant complement component in the circulation), and the large surface area of fHbp occupied in the interaction (2,860±177 Å) [10] which could mask immunogenic epitopes. Recruitment of fH by fHbp to sites where antibody responses are initiated could also reduce immunogenicity due to down regulation of complement activation [20] or lead to anti-fH responses and autoimmune phenomena [21]. Furthermore, it has been suggested that sequestration of fH by pathogens (or indeed by vaccines) could co-opt this regulator from endothelial surfaces and render them susceptible to complement mediated damage [21]. Therefore the overall aim of this work was to define fHbps which are significantly impaired in their ability to bind fH (*i.e. non-functional fHbps*). Our approach was to perform detailed structure:function analyses, and to assess their immunogenicity in a relevant model. Our work identifies novel residues in fHbp from each variant family that substantially affect the interaction with fH. For those fHbps examined as vaccine candidates, we demonstrated that the lack of fH binding did not simply result from a change in their structure. Of note, the distribution of amino acids in fHbp that contribute to fH binding are distinct for proteins from each variant family, despite conservation in the overall atomic structure of the proteins and affinity of their interaction with fH. The V1, V2 and V3 fHbps exhibited similar nanomolar dissociation constants with fH even though we found single amino acid substitutions that significantly enhance binding; this suggests there is selective pressure to maintain a specific strength of fH:fHbp interaction. We also demonstrate that impaired binding of the murine fH to fHbp is not solely due to amino acid differences at the binding site; structural analyses revealed a different orientation of CCPs 6 with 7 in the human and murine molecules that would sterically inhibit interactions with mfH. As a consequence we analysed the immune responses of non-functional fHbps in mice expressing a single chimeric fH consisting of both human (to allow binding to fHbp) and murine (to allow complement regulation) domains. We found that non-functional fHbp retained their immunogenicity and elicited protective immune responses in both transgenic and wild-type mice, supporting the need to evaluate their efficacy in clinical trials.

## Results

### Characterisation of amino acids in fHbp contributing fH interactions and immunogenicity of modified fHbps

**Figure 1:**
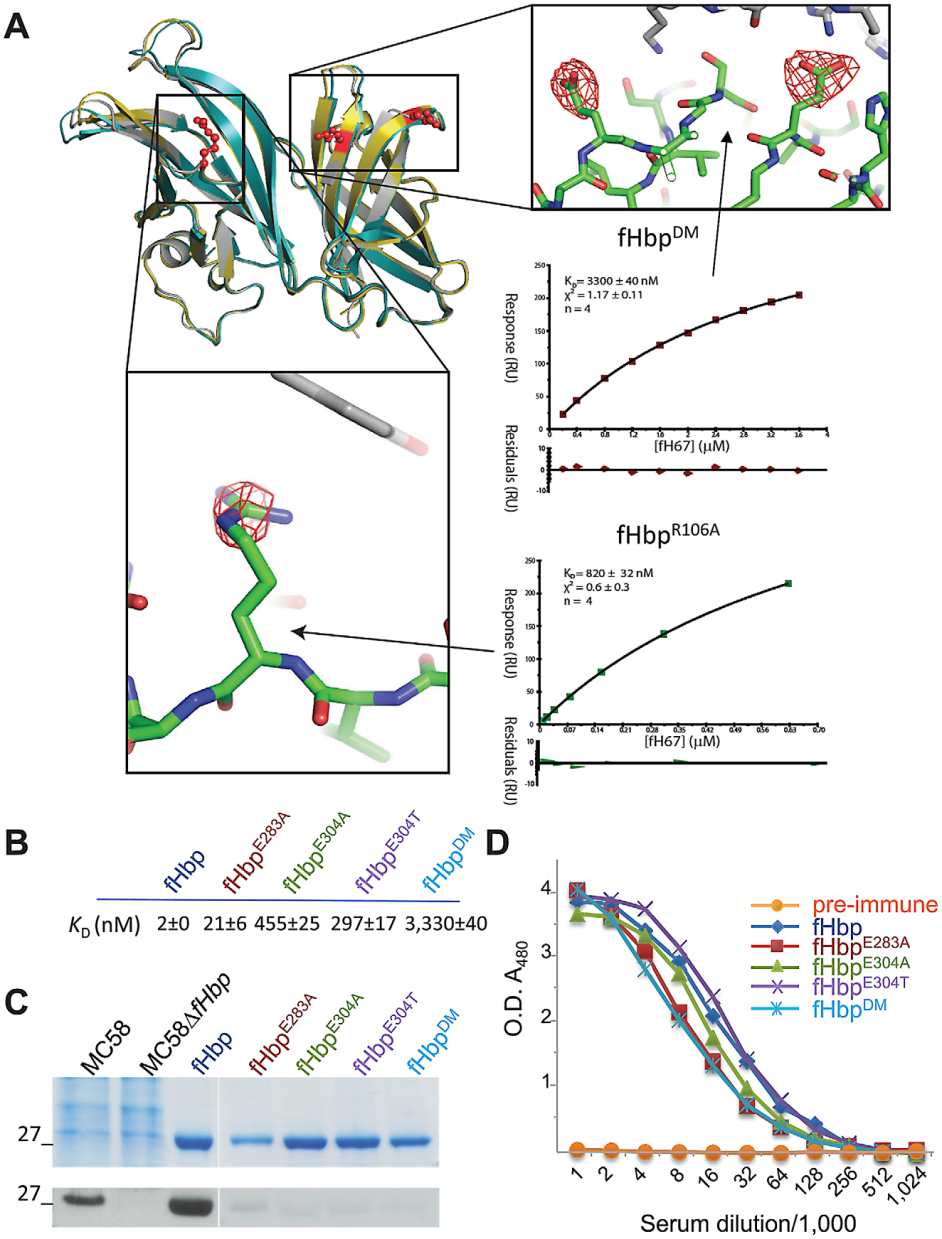
Structure and immunogenicity of V1 fHbps with impaired fH binding. (**A**) Structures of V1 fHbp mutants with reduced binding. Top left shows overlay of cartoon representation of fHbp structures from V1 fHbp (grey), fHbp^DM^ (gold), and fHbp^R106A^ (teal). Zoom boxes show close-ups of modified residues with different densities (F_O_-F_C_) contoured at 4 sigma. Inset panels show typical equilibrium fits for binding to fH_67_, and average *K*_D_ and quality of fit indicators based on four repeats. Binding of V1 fHbp and modified fHbps to fH_67_ by SPR (**B**), and by Coomassie straining (blue bands) or far Western analysis with fH (black bands) to these proteins and lysates from *N. meningitidis* strain MC58 and MC58Δ*fHbp*, the *fHbp* mutant (**C**). (**D**) Antibody titres against V1 fHbps elicited by immunisation with fHbps.

We have shown previously that Ala substitution of two Glu residues (Glu^283^ and Glu^304^) in V1 fHbp (V1.p1, variant group and peptide number, www.neisseria.org), resulting in fHbp^DM^, impairs interactions with fH [10], with data using full length fH (Fig. S1) consistent with results obtained with the binding domain, fH_67_. To determine whether these modifications affect the overall structure of fHbp and thence fH binding, we determined the atomic structure of fHbp^DM^ in complex with fH_67_. The only detectable changes in the fHbp^DM^ structure are loss of the side chains of Glu^283^ and Glu^304^ compared with V1 fHbp (Fig. 1 A, Table S1), even though the dissociation constant (*K*_D_) of fHbp^DM^ with fH_67_ is three orders of magnitude higher than with the wild-type protein assessed by surface plasmon resonance (SPR, Fig. 1B, *K*_D_ for fHbp and fHbp^DM^, 2 nM±0.4 and 3,330 nM±40, respectively). Although substitution of Glu^283^ or Glu^304^ individually (fHbp^E283A^ and fHbp^E304A^, respectively) results in a loss of detectable fH binding by far Western (Fig. 1C), neither residue alone accounts for the profound reduction in affinity observed with fHbp^DM^ (Fig. 1B and Table 1), probably because both residues form independent salt bridges with fH and are therefore both critical for binding. Distinct from Glu^304^ in V1 fHbps, V2 and V3 proteins have Thr in position 304; however this residue cannot substitute for Glu^304^ in V1 fHbp as fHbp^E304T^ also exhibits significantly increased *K*_D_ with fH_67_ (297 nM±17) in comparison with the wild-type protein (Table 1). Furthermore both Glu^283^ and Thr^304^ make independent contributions to the binding to fH of V2 and V3 fhbps albeit to different extents (Table 1).

**Table 1:** Effect of mutations at positions equivalent to fHbp V1 residues 283 and 304 (*i.e.* Thr^304^ in V2 and V3 fHbp) on the *K*_D_ for binding to fH_67_, shown relative to the wild-type proteins.

	Glu^283^Ala	Glu/Thr^304^Ala	DM	304
fHbp V1	7 fold	150 fold	1000 fold	100 fold (Glu→Thr)
fHbp V2	40 fold	10 fold	ND	200 fold (Thr→Glu)
fHbp V3	40 fold	30 fold	70 fold	ND

DM indicates double Ala substitution; ND, not determined.

To determine whether these modified fHbps retain their immunogenicity, wild-type mice were immunised with the recombinant proteins and immune sera assayed for the titres of anti-V1 fHbp antibodies; non-transgenic mice have been used previously to determine the immunogenicity of fHbp [12], [13]. Specific antibody levels were not significantly different from those obtained following immunisation with modified fHbps compared with wild-type V1 fHbp (Fig. 1D); V1 fHbp and the modified proteins all elicited antibody titres in excess of a 1∶32,000 serum dilution. Consistent with this, modified fHbps elicited SBA responses at levels that were not significantly different from the functional, wild-type fHbp. The average SBA titres from at least two independent immunisation experiments (each using pooled sera from at least eight mice) against *N. meningitidis* strain MC58 (which expresses V1.p1 fHbp) were as follows: with sera raised against V1 fHbp, 340; against fHbp^E283A^, 180 (unpaired t test *vs*. V1 fHbp, *p* = 0.17) and fHbp^E304A^, 384 (*p* = 0.913); fHbp^E304T^, 192 (*p* = 0.302); and fHbp^DM^ 170 (*p* = 0.148). Taken together, our results show modification of V1 fHbp at Glu^283^ and/or Glu^304^ does not affect the structure or immunogenicity of the protein, even though these residues contribute significantly to interactions with fH_67_ (Fig. 1B).

### Identification of key amino acids in fHbps from different variant families necessary for high affinity fH interactions

To date, only Glu^283^ and Glu^304^ (Fig. 1) and Arg^106^ [22] in V1 fHbp have been shown to influence interactions with fH; while no data are available for V3 family proteins, a recent report describes three amino acids in V2 fHbp that contribute to the interaction [23] although binding was analysed by ELISA and the affinity of the interaction was not measured. There is relatively low sequence conservation between the fHbp variants in residues buried in the V1 fHbp:fH interface, and of the two Glu residues in V1 fHbp required for fH binding, only Glu^283^ (using V1 numbering, [10]) is conserved in V2 and V3 fHbps. We therefore constructed single and double mutants in the V2 and V3 proteins replacing the equivalents of Glu^283^ and Glu^304^ with Ala, or Glu in the case of position 304. We then determined the effect of these mutations on binding of fH by SPR (Table 1). All modified proteins had reduced capacity to bind fH, but the effect of the mutations at each position differed between the variant families, demonstrating that it is not possible to extrapolate findings from one fHbp variant to others.

Therefore to identify critical amino acids involved in fHbp:fH interactions, we undertook extensive mutagenesis of V1 (V1.p1), V2 (V2.p21) and V3 (V3.p28) fHbps of amino acids that lie in or around the interface of fH in complex with V1 fHbp [10]. In total, 46, 46, and 48 amino acids were individually replaced with Ala in V1, V2, and V3 fHbp respectively, or with the equivalent V1 residue if they were an Ala in the V2 or V3 proteins. We targeted residues at the interface between V1 fHbp and fH_67_ (or equivalent amino acids in V2 and V3 proteins), together with neighbouring amino acids, as well control mutations involving two amino acids, fHbp^K92A^ and fHbp^H248A^, on a region of fHbp opposite to the fH binding site, as well as Leu^171^, a residue buried between the fHbp barrels to probe the effect of a structural alteration. The affinity of the modified fHbps with fH_67_ was determined by SPR with corresponding wild-type fHbps and V1 fHbp^DM^ as controls.

**Figure 2:**
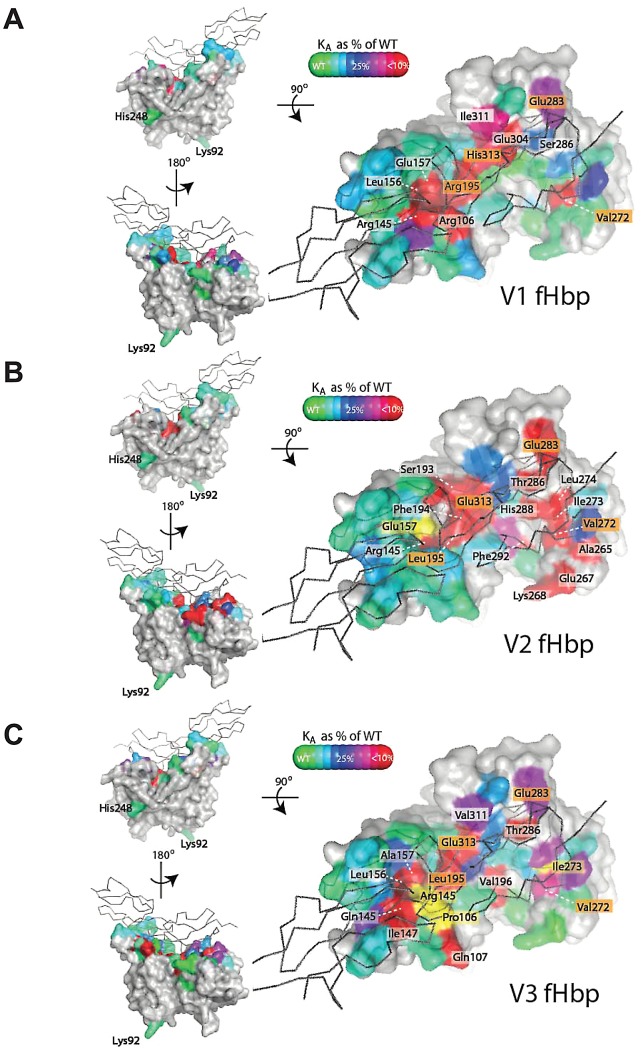
Key residues in fHbp necessary for high affinity interactions with fH. Amino acids of V1, V2 and V3 fHbps (**A**, **B** and **C** respectively, fill representations) superimposed on the structure of V1 fHbp with fH (black sticks) demonstrating the impact of residues on the *K*_D_ as a percentage of results with the corresponding wild-type protein. Substitution of residues coloured yellow increases binding by > fivefold, while amino acids labelled in orange have a significant effect on binding proteins from all three variant groups.

The affinity of parental fHbps for fH_67_ demonstrates that they recognise fH with similar affinities (*K*_D_ for V1 2.2±0.4 nM, V2 1.9±0.2 nM, and V3 2.8±0.0 nM, and Table S2) implying some selection for a specific affinity. The similar affinities of all three variant families for fH is striking as several of our mutations to Ala actually increase the affinity with which fH is bound (Fig. 2A, B and C, and Tables S3, S4 and S5). For instance in V2 fHbp, mutation to Ala at position 157 increases binding by approximately five-fold, whilst mutation at position 106 increases the strength of binding by ∼100-fold in V3 fHbp.

The mutagenesis studies show that the mode of fH binding is conserved between the three families with the same surface of fHbp involved in each variant (Fig. 2A, B and C), with all amino acids in V1, V2 and V3 fHbps that have a substantial contribution to fH binding (*i.e.* Ala substitution causing a ten-fold or greater increase in *K*_D_) located at the interface of V1 fHbp with fH in the crystal structure [10]. In line with this, modifications at several positions (*i.e.* 195, V1^Arg^, V2/V3^Leu^; 272, Val; 283, Glu; 313, V1^His^, V2/V3^Glu^) reduce binding by at least five-fold in all three families; these residues form an extended surface on both *β* barrels of fHbp.

However there are evident differences, with mutation at certain residues having profound effects in the context of a particular variant family, but with little or no effect in others. For instance, in V1 fHbp a key point of contact with fH is seen to be a packing of Arg^106^, Arg^145^, Leu^156^, Glu^157^, Arg^195^ against Tyr^368^ in fH_6_ [10]. This same patch of residues is of some importance in V3, but mutation of only some of these residues affects V2 fHbp binding. For instance, Ala substitution of Arg^106^ or the corresponding residue (*i.e.* Pro^106^ in V3 fHbp) reduces, does not affect, or increases interactions of fH with V1, V2 and V3 proteins respectively (Fig. 2A, B and C and Table S4); our results with V2 fHbp are consistent with others showing no effect of this residue albeit by ELISA [23]. Overall, comparing the amino acids in V1, V2 and V3 fHbps that reduce affinity to fH by over 90% (Fig. 2A, B and C), V2 fHbp is more dependent on contacts within the C-terminal barrel and less susceptible to alteration by mutation within the N-terminal barrel than V1 and V3, where residues critical for high affinity fH binding are spread across the surface of both barrels. This implies that, whilst the overall affinity and mode of interaction are conserved, there is significant variation in which precise fHbp amino acids are critical, indicating a degree of plasticity in the mode of fH binding.

In addition to examining the effect of fHbp sequences on fH binding we also investigated whether the common Tyr^402^His polymorphism in fH_7_ [24] has any significant effect on the interaction with V2 or V3 fHbp. Our previous work showed no significant effect on binding of V1 fHbp and we confirmed that this polymorphism also has no impact on binding of fH_67_ to V2 or V3 proteins (not shown). This suggests that susceptibility to *N. meningitidis* does not contribute to the maintenance of this polymorphism in human populations.

### Structural analysis of V2 and V3 fHbps

**Figure 3:**
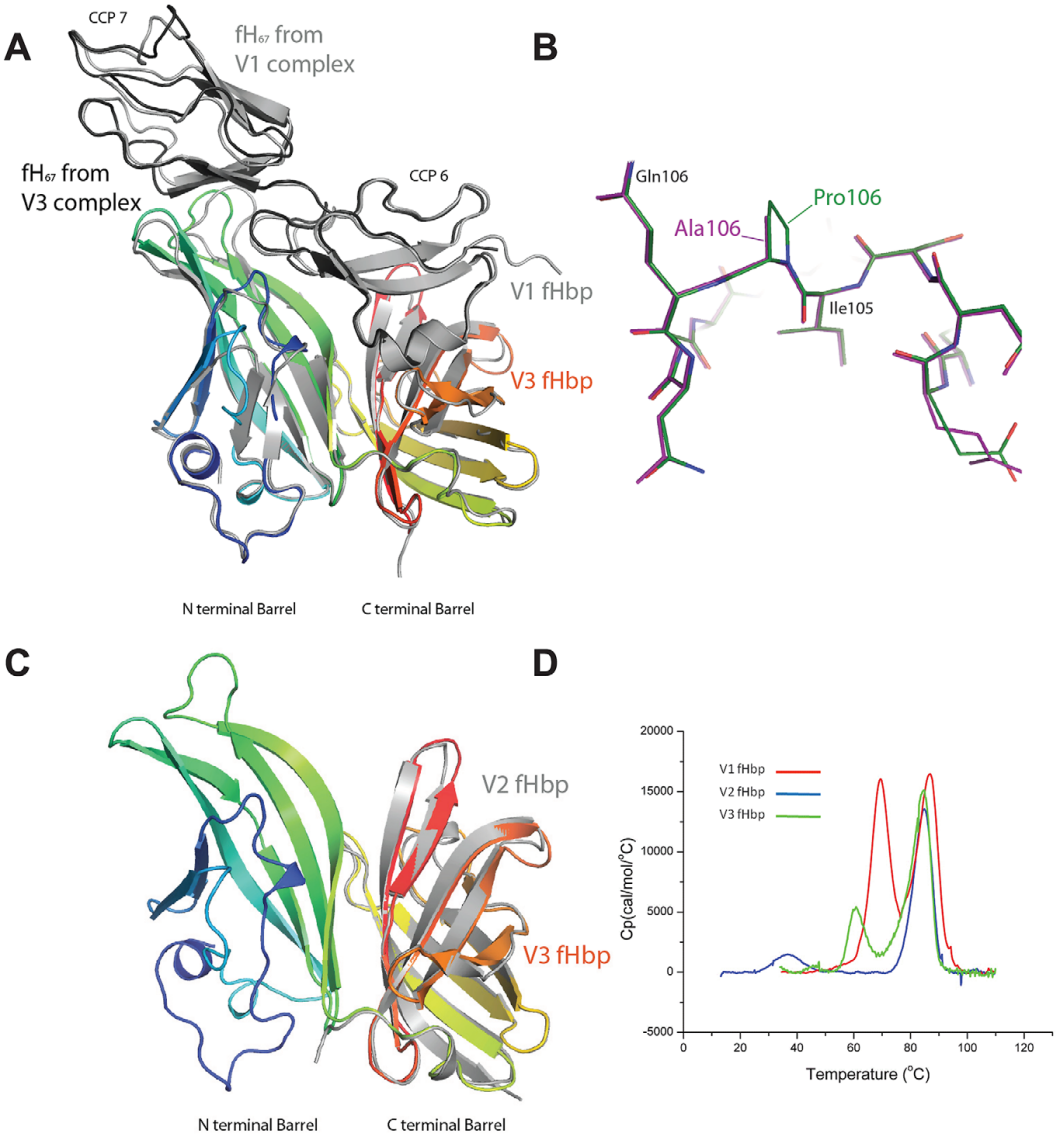
Structures and stability of V2 and V3 fHbps. (**A**) Overlay of V1 and V3 fHbp:fH_67_ complexes shown in a cartoon representation with the V1 complex shown in light grey, and V3 fHbp (rainbow coloured blue at the N-terminus to red at the C-terminus) with fH_67_ from the V3 complex shown in black. (**B**) Overlay of V3 fHbp (stick representation) in the region of residue 106 (magenta) and V3 fHbp^P106A^ (carbon-green, oxygen-red, nitrogen-blue). (**C**) Overlay of V3 fHbp (rainbow coloured blue to red, N- to C-terminus) with the C terminal barrel of V2 fHbp (grey); pictures drawn by PyMol. (**D**) DSC of V1 (red line), V2 (blue line) and V3 (green line) fHbp showing unfolding of the C-terminal barrel at around 80°C for all variants, and the N-terminal barrel at lower temperatures.

To further characterise V2 and V3 fHbps, attempts were made to obtain the atomic structure of these proteins either alone or in complex with fH_67_. The structure of V3 fHbp with fH was solved to a 2.3 Å resolution (Fig. 3A, Table S1). Despite sharing only approximately 60% amino acid identity, the structures of the fHbps are well conserved (Root Mean Square Deviation in all atom positions (RMSD) 0.65 Å) and so is the structure of the complex with fH_67_ (RMSD 0.91 Å) as predicted on the basis of the similarities in distribution of amino acids critical for binding to fH revealed by mutagenesis (Fig. 2). To further understand the contribution of Pro^106^ to V3 fHbp:fH interactions (which markedly increases binding when changed to Ala), we also determined the structure of V3 fHbp^P106A^; with the exception of the change of the side chain, there was no significant alteration in the structure of V3 fHbp^P106A^ compared with the wild-type protein (Fig. 3B), suggesting that the difference is due to a kinetic effect of the conformation in the loop containing this amino acid. Direct comparison of the fHbp V1 and V3 structures in this region did reveal a difference. The presence of an Arg^106^ in V1 fHbp pushes the loop away from fH in order to accommodate the long amino acid side chain. The presence of a Pro in V3 fHbp brings the loop closer to fH and allows the side chain of Gln^107^ in this variant to hydrogen-bond with the fH, consistent with mutation of this residue in V3 reducing binding by 25-fold (Fig. 2C).

Attempts to grow crystals of a complex of V2 with fH_67_ were unsuccessful. Although crystals grown from a mixture of these proteins did diffract, these were found to contain only the C-terminal barrel of the V2 fHbp. This agreed with the observation that V2 fHbp is prone to cleavage to a smaller fragment consistent with the C-terminal barrel alone (not shown). The structure of the V2 fHbp C-terminal barrel is highly conserved with respect to both V1 and V3 fHbp even though it shares under 65% sequence identity with the V1 protein (Fig. 3C). To further examine the apparent instability of the N-terminal *β* barrel of V2 fHbp, differential scanning calorimetry (DSC) was performed on all three variant fHbps (Fig. 3D). The DSC profiles show independent unfolding of the two barrels with the peak representing unfolding of the C-terminal barrel melting at temperatures above 80°C in all three variants. (86.8, 84.9 and 84.5°C for V1, 2 and 3, respectively). In contrast, the N-terminal barrel exhibits highly variable melting at 69.5°C in V1, 60.6°C in V3 and at 36.6°C in V2 fHbp. The much reduced melting point in V2 fHbp suggests that the tendency of this barrel to be cleaved is due to unfolding of the N-terminal barrel, giving access to protease recognition sites within the N-terminal portion.

### Structural basis for the host specificity of fHbp interaction with fH

**Figure 4:**
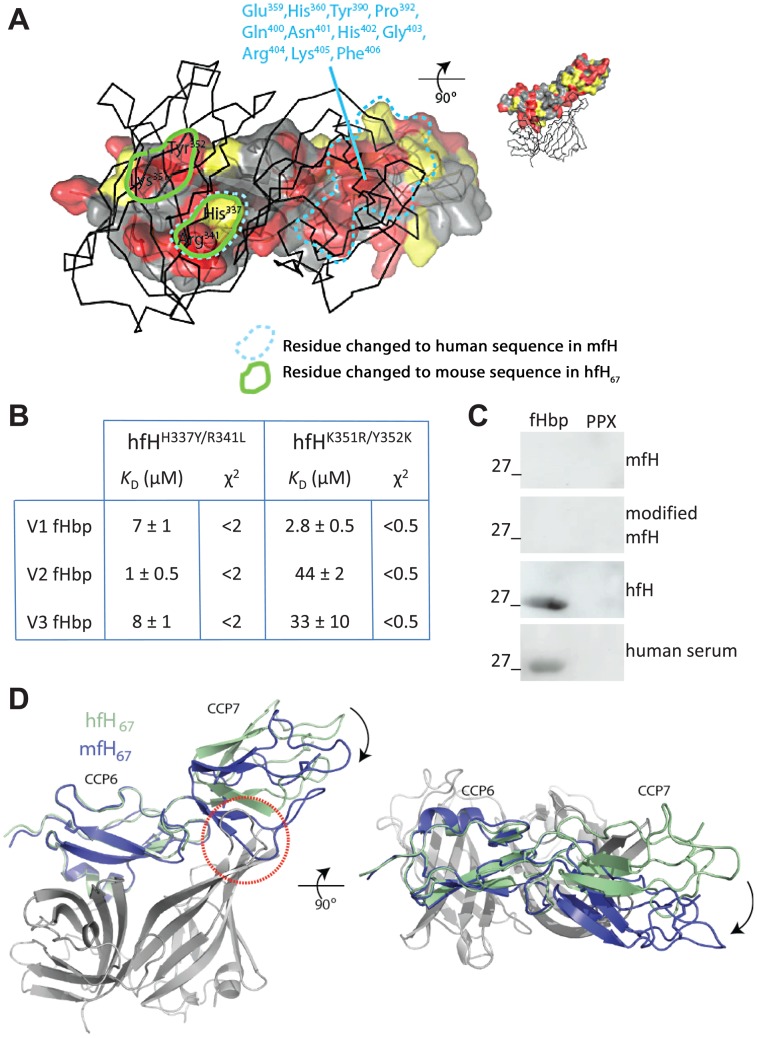
Structural basis for the reduced affinity of mfH with fHbp. (**A**) Cartoon of hfH_67_ viewed from through V1 fHbp (solid line) with amino acids changed in hfH with murine residues (outlined by yellow dashes), and those replaced in mfH with human residues (outlined by light blue dashes). (**B**) SPR analysis of binding of two hfH_67_ mutants each containing two amino acid changes (shown) with fHbps from each variant family. (**C**) Far western analysis of V1 fHbp and a control protein, PPX; blots were overlaid with 5 µg/ml of the recombinant proteins mfH, modified mfH (with 14 humanised amino acids) or hfH, or with human serum (1 in 2000 dilution) as indicated; the sizes of the mol. wt. marker are shown. (**D**) Structure of mfH_67_ (blue ribbon) superimposed on V1 fHbp (white ribbon) and hfH (green ribbon). While fH_6_ from both species are superimposable, the orientation of fH_7_ differs significantly between mfH and hfH (indicated in red dashed circle).

While fHbp binds human fH_67_ with high affinity, murine fH (mfH) interacts but with a *K*_D_>10,000 fold higher than hfH (Fig. S2), meaning there is no significant interaction at serum fH concentrations (150–500 µg/ml, *i.e.* <5 µM). Therefore to develop a physiologically relevant model to test non-functional fHbp vaccines, we sought to define the basis of the binding of fHbp to hfH but not mfH. Alignment of the amino acid sequences of hfH and mfH revealed multiple residues located at the site of interaction with fHbp that differ between the two species (Fig. 4A). Initially, to evaluate the contribution of these residues to interactions with fHbp, we generated two hfH_67_ mutants, each with two amino acids replaced with the equivalent residues from mfH, resulting in hfH^H337Y/R341L^ and hfH^K351R/Y352K^; both modified proteins had significantly reduced affinity for fHbp regardless of variant family (Fig. 4B, for example *K*_D_ for hfH^H337Y/R341L^ and hfH^K351R/Y352K^ with V1 fHbp 7±1 and 2.8±0.5 µM, respectively), demonstrating that amino acid modification of fH can influence binding to fHbp. Therefore, we next humanised 13 residues in mfH that span the region corresponding to the interaction site of hfH with fHbp (Fig. 4A). However this extensive replacement of residues was not sufficient to enable mfH to bind fHbp at appreciable levels as demonstrated by far Western analysis (Fig. 4C); PPX, the meningococcal exo-polyphosphatase was used as a control on blots [25]. To further understand the basis of the lack of interaction, we determined the crystal structure of mfH_67_ (Fig. 4D). The overall CCP folds are conserved despite many sequence differences between the two species throughout the two structures, including the surface where hfH interacts with fHbp. Additionally the arrangement of CCPs 6 and 7 in mfH with respect to each other is distinct from hfH (Fig. 4D), distorting the entire shape of the potential interface with fHbp. This would sterically hinder engagement of mfH with fHbp, providing an explanation for the high *K*_D_ of the fHbp:mfH interaction, and why replacement of multiple amino acids with the human equivalents in mfH did not confer binding.

### Assessment of fHbp-based vaccines in a transgenic model

Therefore to examine the impact of the interaction with fH on the immunogenicity of fHbps, we took advantage of mice lacking endogenous mfH [26], and expressing a transgene encoding a chimeric fH molecule [27]. The chimeric fH consists of mfH CCPs 1–5 and 9–20 (enabling interaction with murine C3b and other complement components), flanking hfH CCPs 6–8 (allowing binding to fHbp, Fig. S3A). The chimeric fH is under the control of the *apoE* promoter to facilitate expression in the liver, the site of endogenous fH synthesis [27]. The chimeric fH effectively regulates the murine complement system; mice have normal C3 levels and do not develop renal disease (Fig. S3B and not shown). Therefore this transgenic model provides a physiologically relevant system to examine the pre-clinical vaccine candidacy of fHbp and its derivatives.

**Figure 5:**
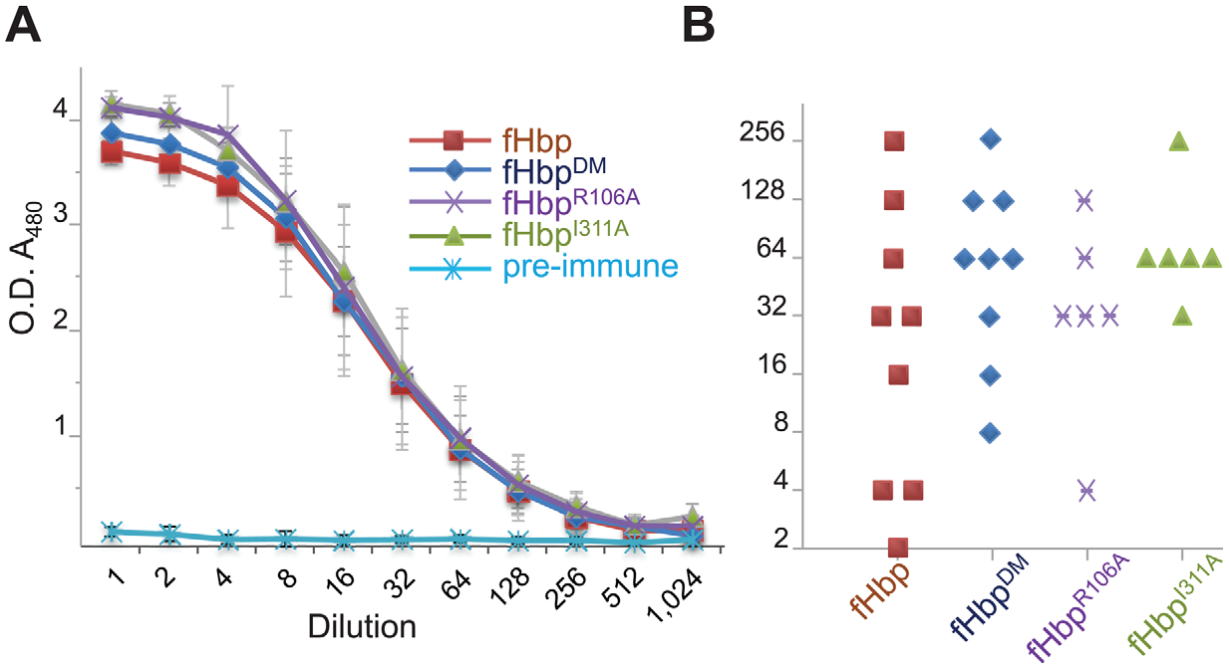
Non-functional fHbps retain their immunogenicity in transgenic mice. (**A**) ELISAs assaying anti-V1 titres elicited in pooled sera following immunisation of transgenic mice with the wild-type protein and non-functional V1 fHbps. (**B**) SBA titres of sera from individual mice immunisation with fHbps.

These mice were used to evaluate the vaccine candidacy of non-functional V1 fHbps compared with V1 fHbp. Further work focussed on the other non-functional mutants, fHbp^R106A^ and fHbp^I311A^; modification of Arg^106^ has been described previously, while Ala substitution of Ile^311^ has the one of the most marked effects on fH_67_ interactions of single amino acid substitutions as demonstrated by SPR (fHbp^I311A^ *K*_D_ with fH_67_, 1.3±0.5 µM). The structure of the co-complex of fHbp^R106A^ with fH_67_ (Fig. 1A) confirmed that there was no significant change in its overall structure. Immunisation of transgenic mice with the non-functional proteins, fHbp^DM^, fHbp^I311A^, and fHbp^R106A^ elicited similar levels of anti-V1 fHbp antibodies as determined by ELISA (Fig. 5A), demonstrating that the non-functional fHbps retain their antigenicity. The non-functional proteins also elicited SBA titres (measured using human complement), that were similar as raised against the wild-type V1 fHbp (Fig. 5B). Therefore non-functional fHbps retain their immunogenicity and elicit protective immune responses.

## Discussion

fHbp is an important virulence factor and a key component of vaccines designed for the prevention of serogroup B *N. meningitidis* infection. Furthermore, fHbp-based vaccines could provide coverage irrespective of serogroup by either combining it with other antigens or using proteins from different variant families [4]. The antigen has been delivered as a recombinant protein in vaccines undergoing Phase II and III clinical trials, but can also be overexpressed in OMV vaccines by genetic modification of strains used for vaccine production [28]. Here we characterised members of the three variant families by identifying amino acids that are critical for fH binding and through structural analysis, to inform future vaccine design and to understand the basis of the interaction of fHbp with fH.

The three variant family fHbps we examined all exhibited nM *K*_D_s with fH_67_, which is lower than for any human ligand of this important complement regulator. Although fHbps have been found in clinical isolates with reduced affinity for fH [29], none have displayed increased binding. Despite this, we were able to identify several single amino acid substitutions that led to a substantial increase in affinity with fH_67_, suggesting that selection may not favour tighter binding, indicating that there could be circumstances when uncoupling of fHbp from fH is beneficial for the bacterium. It is possible that fHbp has other functions [30] which are impaired by the presence of fH. Alternatively disengagement from fH could promote colonisation of different sites in the human host, similar to the way modification of pili facilitates disaggregation of bacteria on the surface of cells [31].

Characterisation of the two Glu residues in V1 fHbp that form salt bridges with fH, and their equivalent residues in V2 and V3 proteins (*i.e.* Thr^304^) indicated that these residues make independent contributions to high affinity fH binding and that different variant family proteins engage fH in distinct ways. To identify residues that are necessary for high affinity fH interactions, we performed extensive Ala substitution mutagenesis to produce a catalogue of amino acids in each variant family that contribute to binding to fH, and could be modified in vaccine design. This adds to the three residues already described for V1 fHbp and for V2 fHbp which are required for high affinity binding, although the affinity of the modified V2 proteins for fH was not reported [23]. Our findings illustrate differences in the precise mechanisms by which fH engages fHbp from different families, even though the same face of fHbp is involved. This is emphasised by the finding that Ala substitution of amino acids at the same position (*i.e.* V1 and V2 Arg^106^, and V3 Pro^106^) have profoundly different effects, markedly reducing, not affecting, or increasing fH affinity for V1, V2 and V3 fHbps, respectively. This demonstrates that it is not possible to extrapolate data from one variant family protein to others. The dramatic increase in tightness of binding on mutating Pro to Ala at this position in V3 probably results from a kinetic effect, suggesting that in the unbound fHbp the loop containing this residue adopts a different conformation which must be refolded into the conformation seen in the complex. It may be that the Pro converts less readily to the structure required for binding than the loop bearing an Ala at this position Despite these distinctions, all amino acids from V1, V2, and V3 fHbps necessary for high affinity binding are located at the interface previously identified in the V1 fHbp:fH co-complex [10].

We determined the first structures of the entire V3 fHbp and the C-terminal *β* barrel of V2 fHbp. There is a striking conservation in the overall structure of the V1 and V3 proteins despite their relatively low level of sequence identity. Although amino acids that contribute to high affinity interactions are grouped in the same regions of these proteins, the precise interactions required to achieve the same affinity and overall interaction with fH differ. Such plasticity could permit the bacterium to alter the fH recognition site for immune evasion whilst retaining the same biological function.

The instability of V2 fHbp and its susceptibility to proteolysis are not desirable in a vaccine antigen, and might explain why it has not been included in any vaccines in clinical trials to date [5]. Such instability is less likely to present an issue in the context of the protein on the exterior of bacteria where interactions with surrounding molecules are likely to stabilise the structure, rendering it competent for binding fH; however it might explain why more C-terminal residues appear to be critical for fH binding compared with fHbp from other variant families. Further work is on-going to define the basis of the instability of V2 fHbp, as there is no obvious explanation for this by molecular modelling using the V1 and V3 structures (not shown).

The use of transgenic mice to study human pathogens has been an important advance in infectious diseases research and prevention. For instance, introducing single amino acid changes into murine molecules [32] or transgenes encoding complete cellular receptors or nutritional sources [33], [34] have allowed the study of human-specific pathogens in rodents. However care must be taken when modifying regulatory factors that govern the activity of complex pathways such as the complement system. We attempted to make minimal changes to mfH within the region that mediates high affinity interactions with fHbp, which would allow binding to the antigen without compromising the important regulatory functions of the molecule. Initial efforts to achieve this by introducing multiple amino acid changes in mfH proved unsuccessful, most likely due to the orientation of CCP 6 with 7 in mfH which would sterically inhibit interactions with fHbp. Therefore, we used a chimeric fH which was humanised through substitution of the CCPs involved in interactions with fHbp together with hfH_8_ in case it induced unforeseen structural changes in fH_7_ [35]. This model provides a physiological assay to evaluate non-functional fHbps, rather than simply introducing an intact human transgene, and was employed to examine the immunogenicity of functional and non-functional fHbps.

Overall there were no substantial differences in the immune responses in transgenic and wild-type mice vaccinated with the same protein; both generated similar levels of IgG and SBA responses against the antigen and relevant strain. Previous work suggests that the immunogenicity of fHbp^DM^ is impaired compared with V1 fHbp [36]. However we found that the structure of this protein is unchanged except for the loss of the side chains of Glu^283/304^, and that it retained its immunogenicity in both transgenic and non-transgenic mice. We also examined the immunogenicity of fHbp^I311A^ which we predict reduces the affinity due to the loss of interactions with the bulkier Ile side chain in the Ala mutant. Previous work indicated that V1 fHbp^R106S^ exhibits a degree of enhanced immunogenicity compared with wild-type fHbp in mice possessing extra copies of hfH as well as endogenous mfH [37]. SBA activity was increased by only a single dilution in mice immunised with the non-functional fHbp compared with the wild-type protein, and the effect was only seen in mice with hfH levels above a certain threshold. However we were unable to replicate this finding either with the corresponding protein, V1 fHbp^R106A^, or with two other non-functional fHbps, fHbp^DM^ and fHbp^I311A^, and did not observe a relationship between fH levels and SBA titres in individual mice (Fig. S4). This is unlikely to result from the hydroxyl side chain in Ser in fHbp^R106S^ compared with fHbp^R106A^ (used here). Potential explanations for these discrepancies in immunogenicity include differences in antigen and adjuvant preparation, immunisation schedules, and the age of mice and their genetic background (C57/Bl6 here *vs*. BALB/c). Furthermore the effects on immunogenicity of the presence of both murine and human fH in a single animal, or an antigen binding hfH (which might not function efficiently in a heterologous environment) are not known.

Any rodent model of immunogenicity has inherent limitations. For instance, both we and others [37] immunised mice with 20 µg of fHbp on each occasion. This is relatively a much higher dose than given to infants in current formulations (50 µg) [5], so the proportion of antigen bound by fH might be significantly lower in rodent than in humans. Additionally the route of immunisation (intraperitoneal in rodent models, subcutaneous in clinical trials) will affect delivery to and the site of immune induction, while results from inbred rodent lines will not be directly applicable to human populations. Despite these reservations, ours and other's findings demonstrate that a series of non-functional, structurally defined fHbps elicit at least equivalent responses to V1 fHbp, and provides proof in principle that these antigens merit evaluation in clinical trials which would provide the only definitive evidence of whether they offer advantages as a vaccine compared with wild-type proteins in terms of safety and immunogenicity.

The efficacy of vaccine antigens can be substantially enhanced by structure based studies to generate non-toxic derivatives of bacterial molecules or antigens with increased efficacy [38]. Here we show that even though V1, 2 and V3 fHbps exhibit remarkably conserved atomic structures, differences in key amino acids necessary for interactions with fH are only revealed by functional studies. Our findings both provide a catalogue of proteins that could be included in the rational development of the next generation of vaccines containing non-functional fHbps, and could be informative about the basis of the diversity in fHbp sequences seen among clinical isolates, and the genetic susceptibility of individuals to meningococcal disease [28].

## Materials and Methods

### Bacterial growth and Western analysis

*N. meningitidis* was grown in 5% CO_2_ on Brain Heart Infusion (BHI) agar plates with Levanthal's supplement, and *Escherichia coli* propagated in LB liquid medium with shaking at 200 r.p.m. or on LB agar plates (1.5% agar wt/vol). Whole cell lysates were prepared of *N. meningitidis* grown overnight on solid media then re-suspended in PBS. The concentration of bacteria was determined by measuring the optical density at 260 nm of bacterial lysates in 1% SDS/0.1 M NaOH [39] and adjusted to 1×10^9^ CFU/ml, and re-suspended with an equal volume of 2× SDS-PAGE loading buffer (100 mM Tris-HCl pH 6.8, 20 µM β-mercaptoethanol, 4% SDS, 0.2% bromophenol blue, 20% glycerol), and boiled for 10 minutes; polyacrylamide gels which were either stained with Coomassie blue or proteins were transferred to nitrocellulose membranes in a Mini Trans-Blot Cell. Membranes were incubated with primary then secondary antibodies diluted in PBS-T and 1% skimmed milk (PBS-TM), which were detected using Amersham ECL Western blot detection method (GE Healthcare). To detect fH binding, blots were incubated in either normal human serum (NHS 1∶100), purified (5 µg/ml) or recombinant fH diluted in PBS-TM for two hours, washed then incubated with goat anti-fH pAb (Quidel, 1 in 2,000); membranes were then incubated with murine anti-goat HRP-conjugated IgG (Sigma, 1 in 10,000).

### Generation of sera and immunologic studies

Female six to eight-week-old BALB/c mice (Charles Rivers, Margate) were immunised with antigens (20 µg) with aluminium hydroxide adsorbed by spinning the mixture for one hour at room temperature. Immunogens were given intraperitoneally (transgenic mice) on days 0, 21 and 35; sera were collected on day 49. In immunisation studies with C57Bl/6 transgenic mice, antigens were given intraperitoneally to twelve to sixteen-weeks-old mice on days 0, 21 and 35, and whole blood collected by terminal anaesthesia and cardiac puncture from the mice on day 49. All procedures were conducted in accordance with Home Office guidelines.

Wells of ELISA plates (Nunc) were coated with V1 fHbp (100 ng) overnight at 4°C, washed, blocked for one hour with 3% normal goat serum diluted in PBS-T, then sera added at a range of dilutions. Binding was detected using goat anti-mouse HRP-conjugated IgG (Dako, 1 in 1, 000) and incubated for one hour at room temperature. The substrate (ONPG, Sigma) was added to wells, the reaction was stopped with 3N HCl, and the A_492_ read with a Multiskan photometer (Thermo Scientific).

For serum bactericidal assays, *N. meningitidis* MC58 was re-suspended in SBA assay buffer (0.1% glucose in PBS) to a final concentration of 5×10^4^ CFU/ml and mixed with an equal volume of human complement. Control wells were also prepared containing bacteria without serum or without complement. Sera was pooled from groups of non-transgenic mice (n>8), and immunisations repeated on two or three occasions for each antigen; for transgenic mice, sera was tested from individual animals. Following incubation, 10 µl from each well was plated onto solid media, and the number of surviving bacteria was determined after overnight growth. The bactericidal activity was expressed as the reciprocal of the highest dilution of sera required to kill more than 50% of bacteria.

### Modification of fHbp and fH

Point mutations in fHbp were introduced by site directed PCR mutagenesis with Roche Expand High Fidelity enzyme or using the QuikChange Site-Directed Mutagenesis Kit (Agilent Technologies) following the manufacturer's protocols, and primers shown in Table S6. His-tagged proteins were expressed in *E. coli* B834 (DE3) cells and isolated using Ni-NTA Magnetic Agarose Beads (Qiagen) following the manufacturer's protocols and dialysed against 50 mM Sodium acetate, pH 4.5. A comparison of the numbering of fHbp amino acids here and by others is shown in Table S7. mfH_67_ was cloned from the full-length *Mus musculus* fH gene into pET-15b expression vector (Novagen) using the following primers. MFH67-For 5′-GGAGATATACCATGGCCTTGAAACCATGTGAATTTCC-3′, and MFH67-Rev 5′-AGCCGGATCCTCGAGTCAGATGCATTTGGGAGGAGG-3′. mfH_67_ was expressed and purified using the method described previously [40]. Crystals were grown from a 11.9 mg/ml solution in a 50% dilution of 0.2 M Ammonium chloride, 0.1 M MES, pH 6.0, 2% PEG 6000 and then cryo-protected in 20% glycerol.

To humanise recombinant mfH, point mutations were introduced in the mouse fH cDNA by site-directed mutagenesis using the QuikChange Multi Site-Directed Mutagenesis kit (Stratagene) according to manufacturer's instructions. Primers used can be found in Table S8. The eukaryote expression vector pCI-Neo (Promega) containing the cDNA of wild-type mfH, humanised mfH or hfH, was used for transient transfection in COS7 cells by using lipofectamine (Invitrogen). Cell supernatants containing the recombinant proteins were collected [41].

C3 and fH levels were measured by ELISA. In brief, C3 levels were quantified using goat anti-mouse C3 and HRP-conjugated goat anti-mouse C3 antibodies (both from MP Biomedicals) as capture and primary antibodies, respectively. The results were quantified by reference to a standard curve generated from acute-phase sera containing a known amount of C3 (Calbiochem). fH levels were measured using goat anti-human fH antibody (ABIN113017, www.antibodies-online.com) and the biotinylated version of the same antibody as capture and primary antibodies, respectively. The results are presented as O.D. values as no reference is available to use as a standard curve for the chimeric protein.

### Large scale protein expression, purification and binding studies

*E. coli* BL21 (DE3) cells with relevant plasmids were grown in liquid medium to an OD A_600_ of 0.4–0.8 then IPTG was added to a final concentration of 1 mM. After four hours, bacteria were harvested and recombinant proteins purified by affinity chromatography with a HisTrap column (GE Healthcare). Proteins were purified with an AKTApurifier (GE Healthcare) by elution with 200 mM imidazole. Further purification was performed by size exclusion chromatography (Superdex S-200). Protein concentrations were estimated by the Bradford assay.

Surface Plasmon Resonance was performed using a Biacore 3000 (GE Healthcare) or ProteOn XPR36 (BioRad). fHbp was immobilized on a CM5 or ProteOn GLM sensor chip and increasing concentrations of fH_67_ were injected over the flow channels at 40 µl/min and allowed to dissociate for 300 seconds. BIAevaluation 3.2 or ProteOn manager software was used to calculate the *K*_D_.

### DSC analysis

DSC experiments were carried out using a VP Capillary DSC (GEHealthcare) using a heating rate of 1°C/min from 30 to 110°C. The V2 sample was repeated from 10 to 110°C when its lower melting event was identified at around 35°C to ensure that this transition was flanked by sufficient baseline to allow analysis. Samples contained 20 uM of each variant in 25 mM Tris pH7.5, 150 mM Na Cl. Samples and buffer were degassed by stirring under vacuum before running. Data analysis was done with the software supplied with the instrument by the manufacturers (Origin version 7.0) with buffer reference subtracted from the sample data and baseline correction.

### Protein structures

The crystals were grown using the sitting drop vapour diffusion method from 400 nl drops prepared using an Oryx Nano robot (Douglas Instruments, UK). V1 fHbp crystals were grown and cryo-protected as described previously (*8*). V2 crystals were grown from a 1∶1 mixture of fH67 and V2.p21 at 10 mg/ml in 30% PEG2KMME, 0.1 M Sodium Acetate pH 4.6, 0.2 M Ammonium sulphate, and cryo-protected with 10% PEG 400. The dataset was collected on beamline ID29 at ESRF. For V3 crystals, concentrations of 13.6 and 15.2 mg/ml were used for V3 fHbp and fHbp^P106A^. Both grew in 0.2 M imidazole pH 6, 20% PEG 4000, and were cryo-protected with 15% ethylene glycol and 85% mother liquor. Data were obtained on I04 (for fHbp^P106A^) at Diamond Light Source (Harwell, England) and ID29 at ESRF (Grenoble, France, for V2 and V3 fHbp). Diffraction data were processed with XDS and SCALA [42] from within the xia2 data-processing suite [43]. Structures were solved by molecular replacement with CCP4 [44] and Phaser [45], built using CCP4-Buccaneer [46] and refined and rebuilt iteratively using autoBUSTER [47] and Coot [48].

### Ethics statement

All work with animals was conducted in accordance with the United Kingdom Home Office guidelines under relevant project licences. Work was approved by the Riverside Local Ethics Committee and performed under licence number PPL 70/6960 awarded to CMT.

## Supporting Information

Figure S1 Binding of full length fH to V1.1 fHbp and the non-functional protein, fHbp^DM^.
(PPTX)

Figure S2 Dose response curve of mfH_67_ binding V1 fHbp.
(PPTX)

Figure S3 Binding of chimeric fH to fHbp by far Western, and the relationship between fH and C3 levels in transgenic mice.
(PPTX)

Figure S4 Relationship between fH and SBA in transgenic mice.
(PPTX)

Table S1 X-ray data and refinement statistics.
(DOCX)

Table S2 Affinity of interactions between fHbps and fH_67_.
(DOCX)

Table S3 Layout of CHIP used for analysis of V1 fHbp mutants and their *K*_D_ values.
(PDF)

Table S4 Layout of CHIP used for analysis of V2 fHbp mutants and their *K*_D_ values.
(PDF)

Table S5 Layout of CHIP used for analysis of V3 fHbp mutants and their *K*_D_ values.
(PDF)

Table S6 Primers used for mutagenesis of V1, V2 and V3 fHbp.
(PDF)

Table S7 Numbering of amino acids in fHbp.
(DOCX)

Table S8 Primers used to modify mfH.
(DOC)
