# Correction: Design and Evaluation of Meningococcal Vaccines through Structure-Based Modification of Host and Pathogen Molecules

**DOI:** 10.1371/annotation/3e7e6415-fb12-4a87-89e6-f87d2e800ba8

**Published:** 2013-01-17

**Authors:** Steven Johnson, Lionel Tan, Stijn van der Veen, Joseph Caesar, Elena Goicoechea De Jorge, Rachel J. Harding, Xilian Bai, Rachel M. Exley, Philip N. Ward, Nicola Ruivo, Kaushali Trivedi, Elspeth Cumber, Rhian Jones, Luke Newham, David Staunton, Rafael Ufret-Vincenty, Ray Borrow, Matthew C. Pickering, Susan M. Lea, Christoph M. Tang

Some information is missing from the Funding statement. The following should be appended to the end of the first sentence:

"and EUCLIDS (Grant number: FP7 GA no. 279185). Work in SML’s laboratory is funded by the Oxford Martin School."

Also, Supplementary Tables S3, S4 and S5 are formatted incorrectly, with a page missing for each. The correct files can be found below. Please view Table S3 here:

Please view Table S4 here:

Please view Table S5 here:
