# Supplementary material for: Design and Evaluation of Meningococcal Vaccines through Structure-Based Modification of Host and Pathogen Molecules
Source: PLoS Pathog. 2012 Oct 25;8(10):e1002981. doi: 10.1371/journal.ppat.1002981 (PMC3486911; doi:10.1371/journal.ppat.1002981)
Supplement: Figure S1 — Binding of full length fH to V1.1 fHbp and the non-functional protein, fHbpDM. (PPTX) [file ppat.1002981.s001.pptx]

## Slide 1
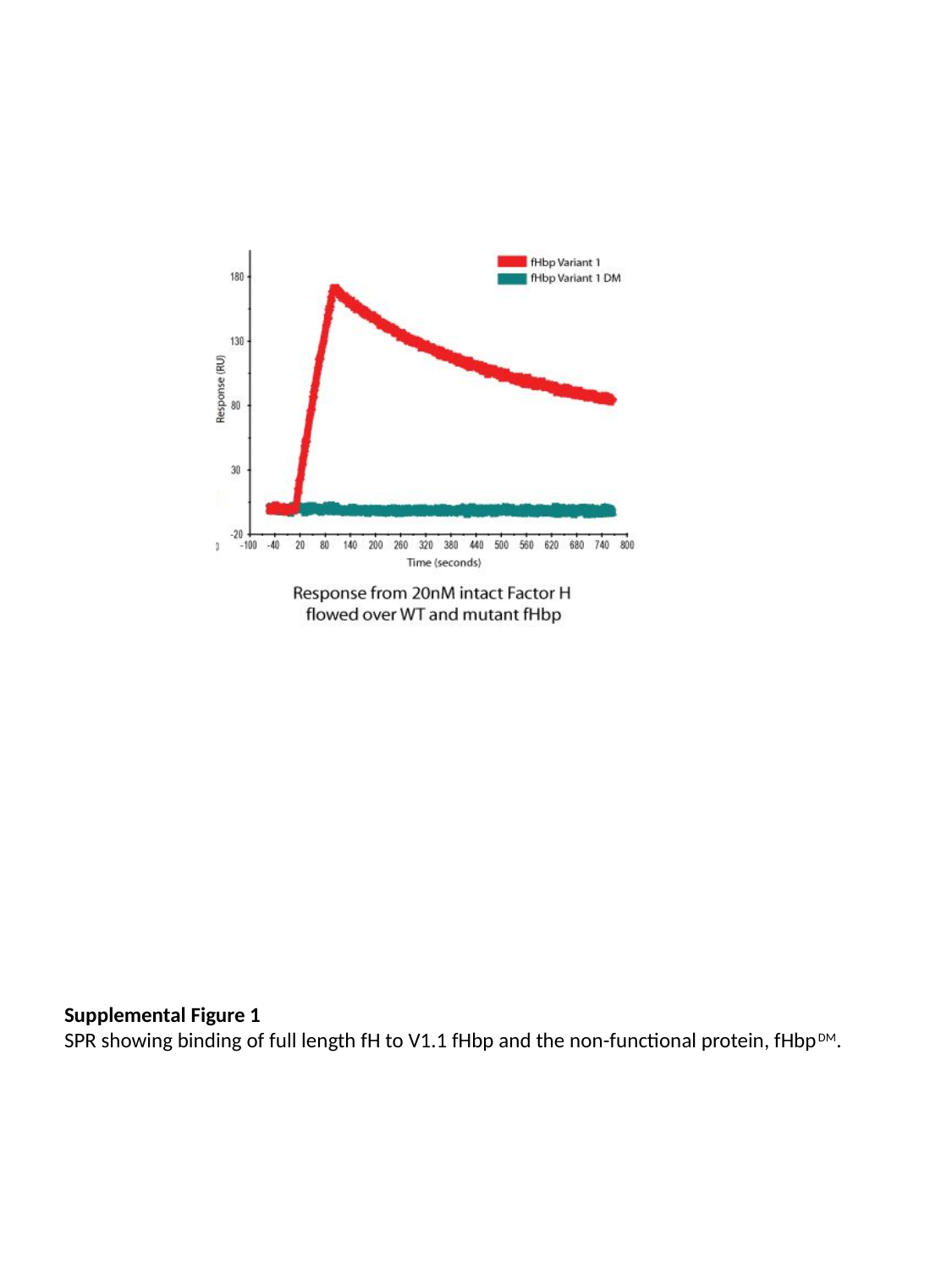

Supplemental Figure 1
SPR showing binding of full length fH to V1.1 fHbp and the non-functional protein, fHbpDM.
