# Supplementary material for: Design and Evaluation of Meningococcal Vaccines through Structure-Based Modification of Host and Pathogen Molecules
Source: PLoS Pathog. 2012 Oct 25;8(10):e1002981. doi: 10.1371/journal.ppat.1002981 (PMC3486911; doi:10.1371/journal.ppat.1002981)
Supplement: Figure S2 — Dose response curve of mfH67 binding V1 fHbp. (PPTX) [file ppat.1002981.s002.pptx]

## Slide 1
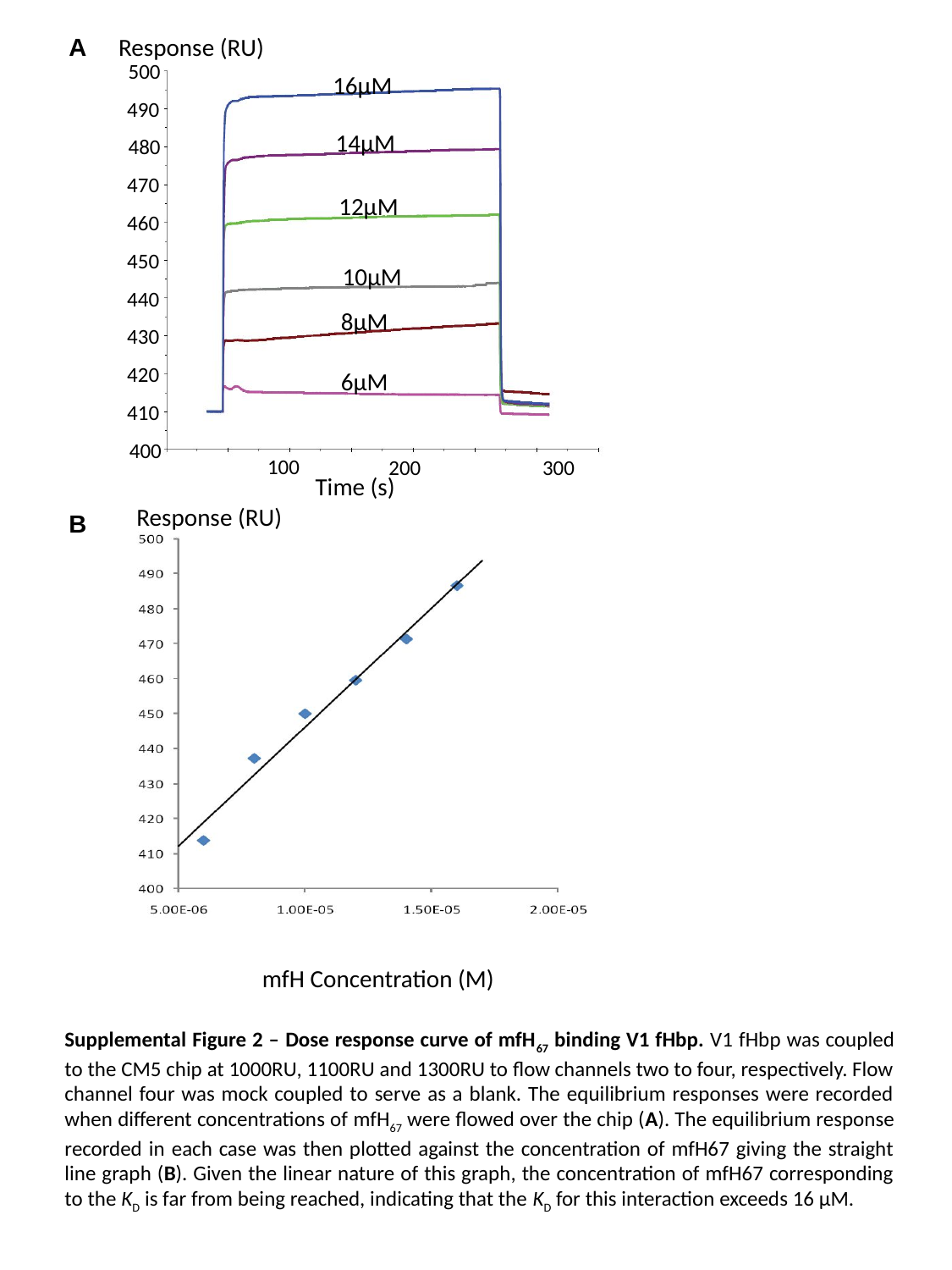

A
Response (RU)
500
16µM
490
14µM
480
470
12µM
460
450
440
8µM
430
420
6µM
410
400
100
300
200
Time (s)
10µM
10µM
Response (RU)
B
mfH Concentration (M)
# Supplemental Figure 2 – Dose response curve of mfH67 binding V1 fHbp. V1 fHbp was coupled to the CM5 chip at 1000RU, 1100RU and 1300RU to flow channels two to four, respectively. Flow channel four was mock coupled to serve as a blank. The equilibrium responses were recorded when different concentrations of mfH67 were flowed over the chip (A). The equilibrium response recorded in each case was then plotted against the concentration of mfH67 giving the straight line graph (B). Given the linear nature of this graph, the concentration of mfH67 corresponding to the KD is far from being reached, indicating that the KD for this interaction exceeds 16 µM.
