# Supplementary material for: Design and Evaluation of Meningococcal Vaccines through Structure-Based Modification of Host and Pathogen Molecules
Source: PLoS Pathog. 2012 Oct 25;8(10):e1002981. doi: 10.1371/journal.ppat.1002981 (PMC3486911; doi:10.1371/journal.ppat.1002981)
Supplement: Figure S3 — Binding of chimeric fH to fHbp by far Western, and the relationship between fH and C3 levels in transgenic mice. (PPTX) [file ppat.1002981.s003.pptx]

## Slide 1
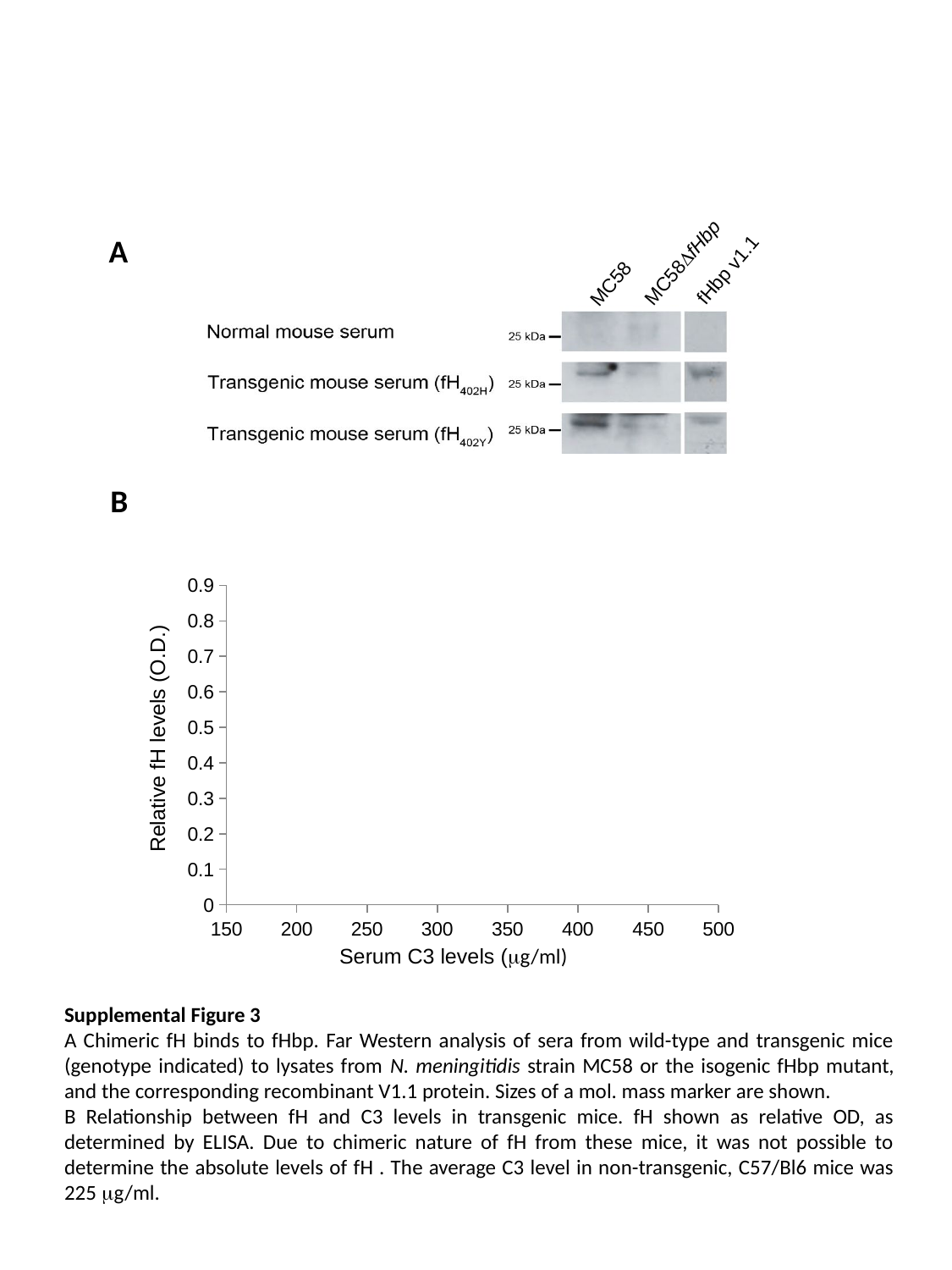

A
MC58DfHbp
fHbp v1.1
MC58
B
### Chart
| Category | |
|---|---|Relative fH levels (O.D.)
Serum C3 levels (mg/ml)
Supplemental Figure 3
A Chimeric fH binds to fHbp. Far Western analysis of sera from wild-type and transgenic mice (genotype indicated) to lysates from N. meningitidis strain MC58 or the isogenic fHbp mutant, and the corresponding recombinant V1.1 protein. Sizes of a mol. mass marker are shown.
B Relationship between fH and C3 levels in transgenic mice. fH shown as relative OD, as determined by ELISA. Due to chimeric nature of fH from these mice, it was not possible to determine the absolute levels of fH . The average C3 level in non-transgenic, C57/Bl6 mice was 225 mg/ml.
