# Supplementary material for: Design and Evaluation of Meningococcal Vaccines through Structure-Based Modification of Host and Pathogen Molecules
Source: PLoS Pathog. 2012 Oct 25;8(10):e1002981. doi: 10.1371/journal.ppat.1002981 (PMC3486911; doi:10.1371/journal.ppat.1002981)
Supplement: Figure S4 — Relationship between fH and SBA in transgenic mice. (PPTX) [file ppat.1002981.s004.pptx]

## Slide 1
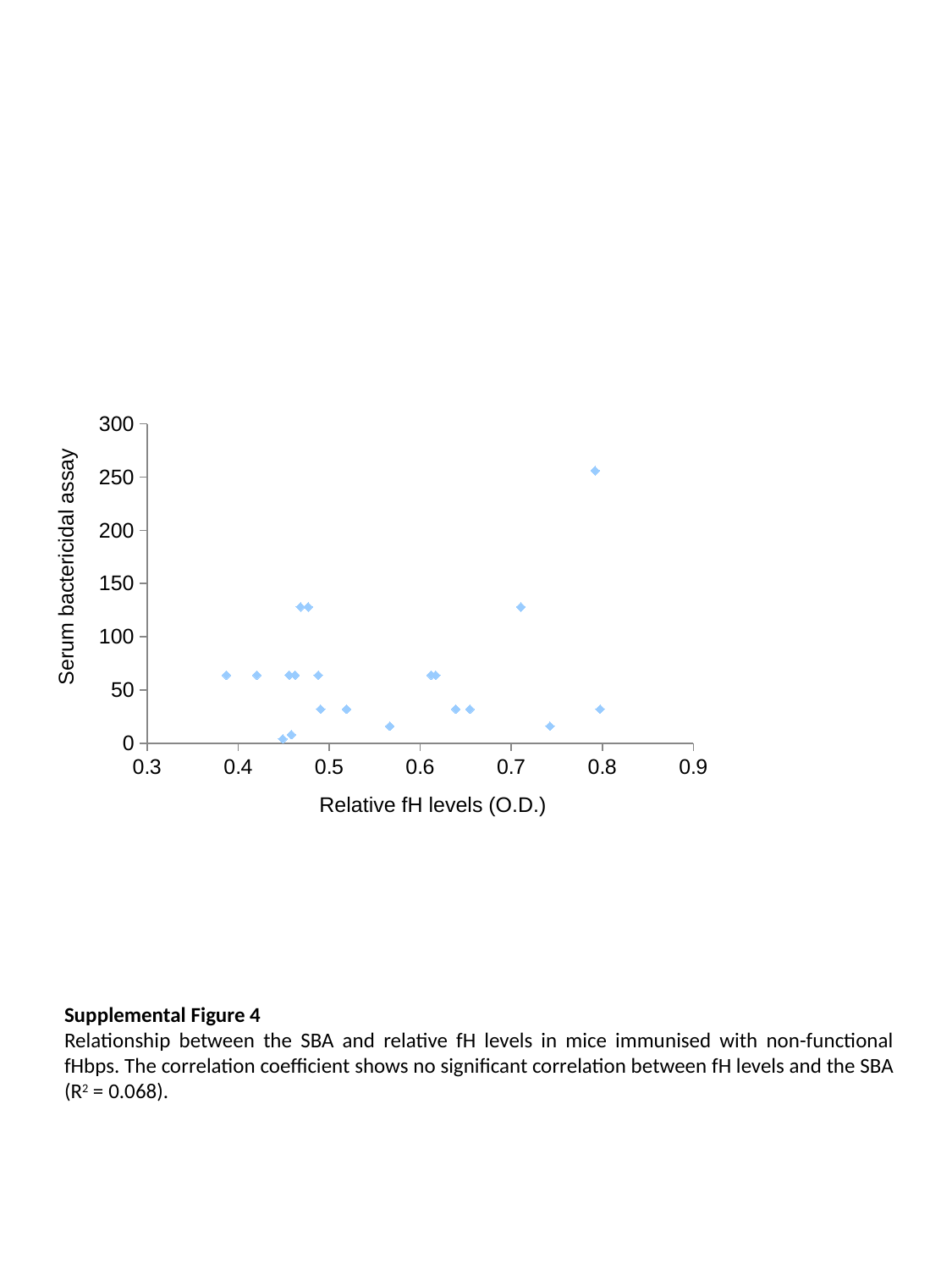

### Chart
| Category | SBA |
|---|---|Serum bactericidal assay
Relative fH levels (O.D.)
Supplemental Figure 4
Relationship between the SBA and relative fH levels in mice immunised with non-functional fHbps. The correlation coefficient shows no significant correlation between fH levels and the SBA (R2 = 0.068).
