# Supplementary material for: Design and Evaluation of Meningococcal Vaccines through Structure-Based Modification of Host and Pathogen Molecules
Source: PLoS Pathog. 2012 Oct 25;8(10):e1002981. doi: 10.1371/journal.ppat.1002981 (PMC3486911; doi:10.1371/journal.ppat.1002981)
Supplement: Table S1 — X-ray data and refinement statistics. (DOCX) [file ppat.1002981.s005.docx]

|  | V1 fHbp^DM^ fH_67_ | V1 fHbp^R106A^ fH_67_ | V3 fHbp/ fH_67_ | V3 fHbp^P106A^ fH_67_ | V2 fHbp (C-terminal barrel) | mfH_67_ |
| --- | --- | --- | --- | --- | --- | --- |
| Space Group | C2 | C2 | P4_1_2_1_2 | C222_1_ | P2_1_2_1_2_1_ | P2_1_2_1_2_1_ |
| Unit Cell Parameters (Å) | a=187.4, b=53.3, c=130.1, β=117.7 | a=185.3, b=54.0, c=129.6, β=118.0 | a=b=57.1, c=363.1 | a=78.1, b=83.5, c=361.4 | a=74.8, b=75.9, c=40.9 | a=29.6, b=34.6, c=107.4 |
| Resolution (Å) | 90.05-2.80 (2.96-2.80 | 114.4-2.4 (2.53-2.4) | 56.4-2.31 (2.38-2.31) | 180-2.85 (2.92-2.85) | 60.0 -2.06 (2.18-2.06) | 35.79-1.58 (1.58-1.58) |
| No. Unique Reflections (Criteria for reflection) | 26592 | 43520 | 27284 | 28212 | 14737 | 15809 |
| R_merge_ | 0.043 (0.170) | 0.064 (0.318) | 0.160 (0.665) | 0.13 (0.71) | 0.045 (0.193) | 0.035 (0.477) |
| [I/σ(I)] | 16.7 (4.3) | 11.8 (3.0) | 10.5 (3.6) | 9.6 (2.7) | 26.0 (6.8) | 28.7 (3.2) |
| Completeness (%) | 94.1 (70.5) | 97.3 (86.1) | 99.9 (98.6) | 99.9 (98.5) | 98.5 (91.3) | 99.8 (99.7) |
| Multiplicity | 2.4 (2.3) | 3.1 (2.3) | 12.8 (13.7) | 7.0 (6.6) | 5.9 (4.1) | 6.5 (6.4) |
| *R*_work_ / *R*_free_ | 0.21 / 0.23 | 0.20/0.23 | 0.22/0.25 | 0.25/0.26 | 0.18/0.21 | 0.19/0.22 |
| No. atoms |  |  |  |  |  |  |
| Protein | 8461 | 8391 | 3805 | 7500 | 1895 | 1950 |
| Ligand/ion | 210 | 16 | 4 | 0 | 45 | 4 |
| Water | 134 | 826 | 204 | 6 | 120 | 129 |
| *Mean B*-factors (Å^2^) | 66.5 | 40.2 | 54.9 | 56.7 | 31.3 | 25.5 |
| R.m.s. deviations |  |  |  |  |  |  |
| Bond lengths (Å) | 0.007 | 0.007 | 0.008 | 0.007 | 0.01 | 0.009 |
| Bond angles (°) | 1.03 | 0.99 | 1.07 | 1.07 | 1.12 | 1.07 |
| Ramachandran plot analysis |  |  |  |  |  |  |
| Most favoured regions (%) | 96.1 | 97.3 | 98.3 | 97.6 | 100 | 98.4 |
| Disallowed regions (%) | 0.4 | 0 | 0 | 0 | 0 | 0 |
| PDB Code | 4aye | 4ayd | 4ayi | 4aym | 4ayn | 2yby |

**Supplemental Table 1 – X-ray Data and Refinement Statistics**
