# Supplementary material for: Design and Evaluation of Meningococcal Vaccines through Structure-Based Modification of Host and Pathogen Molecules
Source: PLoS Pathog. 2012 Oct 25;8(10):e1002981. doi: 10.1371/journal.ppat.1002981 (PMC3486911; doi:10.1371/journal.ppat.1002981)
Supplement: Table S2 — Affinity of interactions between fHbps and fH67. (DOCX) [file ppat.1002981.s006.docx]

**Supplemental Table 2 Affinity of interactions between fHbps and fH_67_**

| Protein | k_on_ (M^-1^s^-1^) | k_off_ (s^-1^) | *K*_D_ (nM) | χ^2^ | n |
| --- | --- | --- | --- | --- | --- |
| V1 fHbp | 3.0e6 ± 1.2 | 6.3e-3 ± 2.3 | 2.2 ± 0.4 | 2.5 ± 1.5 | 9 |
| V2 fHbp | 1.7e6 ± 0.5 | 2.9e-3 ± 0.8 | 1.9 ± 0.2 | 1.2 ± 0.1 | 5 |
| V3 fHbp | 1.6e6 ± 0.6 | 4.5e-3 ± 1.7 | 2.8 ± 0.0 | 3.6 ± 1.2 | 3 |

n, number of assays
