# Supplementary material for: Design and Evaluation of Meningococcal Vaccines through Structure-Based Modification of Host and Pathogen Molecules
Source: PLoS Pathog. 2012 Oct 25;8(10):e1002981. doi: 10.1371/journal.ppat.1002981 (PMC3486911; doi:10.1371/journal.ppat.1002981)
Supplement: Table S3 — Layout of CHIP used for analysis of V1 fHbp mutants and their K D values. (PDF) [file ppat.1002981.s007.pdf]

Supplemental Table 3 Layout of CHIP used for analysis of V1 fHbp mutants and their K_D values

Mutant number layout

	L1	L2	L3	L4	L5	L6
A1	Mut25	32	38	44	WT	
A2	26	33	39	45	WT	
A3	27	34	40	46	WT	
A4	28	35	41	47	Lys306	
A5	29	36	42	48	Lys306	
A6	31	37	43	WT	Lys306	

Actual mutant layout

	L1	L2	L3	L4	L5	L6
A1	Lys191	Asp262	Ile273	Ser302	WT	
A2	Gln193	Lys264	Ser274	Lys306	WT	
A3	Phe194	Pro265	Ser286	Ile311	WT	
A4	Arg195	Asp266	Ser288	His313	Lys306	
A5	Ile196	Lys268	Leu289	FH67Y402	Lys306	
A6	His203	Val272	Phe292	V1WT	Lys306	

Amount FHbp bound

	L1	L2	L3	L4	L5	L6
A1	4910	4950	5310	4930	5210	
A2	4900	5590	4570	4010	5180	
A3	4620	4410	4920	4350	5080	
A4	4590	4760	4870	5110	4990	
A5	4620	5360	5070	310	4980	
A6	4460	4570	4400	5110	4990	

Run1 Kd

	L1	L2	L3	L4	L5	L6
A1	4.50E-09	4.40E-09	4.00E-09	1.20E-09	2.30E-09	
A2	5.30E-09	1.30E-08	8.70E-09	4.20E-09	2.30E-09	
A3	6.30E-09	3.10E-09	9.50E-09	1.90E-08	2.40E-09	
A4	7.50E-08	3.10E-09	2.80E-09	2.20E-08		
A5	1.60E-09	3.30E-09	1.70E-09			
A6	2.00E-09	3.40E-08	7.70E-09	3.20E-09		

Run2 Kd

	L1	L2	L3	L4	L5	L6
A1	3.80E-09	3.70E-09	2.90E-09	7.80E-10	2.40E-09	
A2	4.20E-09	1.10E-08	9.30E-09	4.20E-09	2.30E-09	
A3	6.00E-09	2.50E-09	9.30E-09	2.30E-08	2.50E-09	
A4	3.60E-07	2.80E-09	2.70E-09	2.40E-08		
A5	1.40E-09	2.30E-09	1.60E-09			
A6	1.40E-09	3.30E-08	6.50E-09	2.40E-09		

Run1 Chi2

	L1	L2	L3	L4	L5	L6
A1	23	15	7	24	4.3	
A2	18	7	10	12	4.2	
A3	4	27	8	2	7	
A4	1	22	22	8		
A5	21	21	2			
A6	30	2	6	18		

Run2 Chi2

	L1	L2	L3	L4	L5	L6
A1	7.4	4.5	3	0.6	3.5	
A2	6.7	3.3	6.9	3.7	3.8	
A3	2.5	11	7.8	1.3	5.5	
A4	1.5	15	14	8.5		
A5	8.9	2	1.7			
A6	10.6	2.2	4.9	6		

Fold Change

	L1	L2	L3	L4	L5	L6
A1	1.73	1.69	1.44	0.41	0.98	
A2	1.98	5.00	3.75	1.75	0.96	
A3	2.56	1.17	3.92	8.75	1.02	
A4	90.63	1.23	1.15	9.58	0.00	
A5	0.63	1.17	0.69	0.00	0.00	
A6	0.71	13.96	2.96	1.17	0.00	

Average Kd

	L1	L2	L3	L4	L5	L6
A1	4.15E-09	4.05E-09	3.45E-09	9.9E-10	2.35E-09	
A2	4.75E-09	1.2E-08	9E-09	4.2E-09	2.3E-09	
A3	6.15E-09	2.8E-09	9.4E-09	2.1E-08	2.45E-09	
A4	2.175E-07	2.95E-09	2.75E-09	2.3E-08	0	
A5	1.5E-09	2.8E-09	1.65E-09	0	0	
A6	1.7E-09	3.35E-08	7.1E-09	2.8E-09	0	

