# Supplementary material for: Design and Evaluation of Meningococcal Vaccines through Structure-Based Modification of Host and Pathogen Molecules
Source: PLoS Pathog. 2012 Oct 25;8(10):e1002981. doi: 10.1371/journal.ppat.1002981 (PMC3486911; doi:10.1371/journal.ppat.1002981)
Supplement: Table S4 — Layout of CHIP used for analysis of V2 fHbp mutants and their K D values. (PDF) [file ppat.1002981.s008.pdf]

Supplemental Table 4 Layout of CHIP used for analysis of V2 fHbp mutants and their K_D values

Mutant number layout

	L1	L2	L3	L4	L5	L6
A1	V2WT	28	34	40	V1WT	Blank
A2	V2mut24	29	35	41	V1WT	Blank
A3	25	30	36	43	48	Blank
A4	26	31	37	44	V3WT	Blank
A5	27	32	38	45	V3WT	Blank
A6	V1WT	33	39	47	V2WT	Blank

Actual mutant layout

	L1	L2	L3	L4	L5	L6
A1	V2WT	Val196	Glu267	Thr286	V1WT	
A2	Gln191	Leu199	Lys268	His288	V1WT	
A3	Ser193	His203	Val272	Phe292	Glu313	
A4	Phe194	Glu262	Ile273	Ser302	V3WT	
A5	Leu195	Lys264	Leu274	Thr304	V3WT	
A6	V1WT	Ala265Pro	Glu283	Val311	V2WT	

Amount FHbp bound

	L1	L2	L3	L4	L5	L6
A1	1240	3950	70	600	2310	
A2	3900	3820	1600	165	1290	
A3	750	4000	430	25	870	
A4	550	3850	25	1980	740	
A5	3800	3200	550	1300	1120	
A6	0??	310	120	2100	850	

Run1 Kd

	L1	L2	L3	L4	L5	L6
A1	2.30E-09	3.20E-08	NBD	NBD	2.70E-09	
A2	2.70E-09	1.50E-08	2.20E-09	NBD	2.70E-09	
A3	NBD	2.80E-09	NBD	NBD	NBD	
A4	NBD	5.00E-09	NBD	4.40E-09	2.80E-09	
A5	NBD	8.60E-09	NBD	8.50E-09	2.90E-09	
A6	3.10E-09	NBD	NBD	7.90E-09	1.80E-09	

Run2 Kd

	L1	L2	L3	L4	L5	L6
A1	2.00E-09	1.40E-08	NBD	NBD	2.70E-09	
A2	2.50E-09	1.60E-08	2.30E-09	NBD	2.70E-09	
A3	NBD	2.50E-09	NBD	NBD	NBD	
A4	NBD	5.60E-09	NBD	3.70E-09	2.80E-09	
A5	NBD	9.50E-09	NBD	7.90E-09	3.00E-09	
A6	2.60E-09	NBD	NBD	7.00E-09	1.90E-09	

Run1 Chi2

	L1	L2	L3	L4	L5	L6
A1	1.2	0.8	NBD	NBD	5.5	
A2	5.6	12	4.4	NBD	6.8	
A3	NBD	5.6	NBD	NBD	NBD	
A4	NBD	5.2	NBD	2.4	3.4	
A5	NBD	4.1	NBD	1.5	4.5	
A6	1.1	NBD	NBD	1.8	1.2	

Run2 Chi2

	L1	L2	L3	L4	L5	L6
A1	1.3	0.7	NBD	NBD	5.9	
A2	5.3	9.3	2.6	NBD	5.7	
A3	NBD	4.5	NBD	NBD	NBD	
A4	NBD	3.6	NBD	2.6	4.6	
A5	NBD	3.1	NBD	1.3	5.9	
A6	1	NBD	NBD	1.7	1.2	

Fold Change

	L1	L2	L3	L4	L5	L6
A1	1.08	11.50	NBD	NBD	1.35	
A2	1.30	7.75	1.13	NBD	1.35	
A3	NBD	1.33	NBD	NBD	NBD	
A4	NBD	2.65	NBD	2.03	1.40	
A5	NBD	4.53	NBD	4.10	1.48	
A6	1.43	NBD	NBD	3.73	0.93	

Average Kd

	L1	L2	L3	L4	L5	L6
A1	2.15E-09	2.3E-08	NBD	NBD	2.7E-09	
A2	2.6E-09	1.55E-08	2.25E-09	NBD	2.7E-09	
A3	NBD	2.65E-09	NBD	NBD	NBD	
A4	NBD	5.3E-09	NBD	4.05E-09	2.8E-09	
A5	NBD	9.05E-09	NBD	8.2E-09	2.95E-09	
A6	2.85E-09	NBD	NBD	7.45E-09	1.85E-09	

V1 equivalent

	L1	L2	L3	L4	L5	L6
A1	V2WT	Ile196	Gly267	Ser286	V1WT	
A2	Lys191	Ile199	Lys268	Ser288	V1WT	
A3	Gln193	His203	Val272	Phe292	His313	
A4	Phe194	Asp262	Ile273	Ser302	V3WT	
A5	Arg195	Lys264	Ser274	Glu304	V3WT	
A6	V1WT	Pro265	Glu283	Ile311	V2WT	
