# Supplementary material for: Design and Evaluation of Meningococcal Vaccines through Structure-Based Modification of Host and Pathogen Molecules
Source: PLoS Pathog. 2012 Oct 25;8(10):e1002981. doi: 10.1371/journal.ppat.1002981 (PMC3486911; doi:10.1371/journal.ppat.1002981)
Supplement: Table S5 — Layout of CHIP used for analysis of V3 fHbp mutants and their K D values. (PDF) [file ppat.1002981.s009.pdf]

Supplemental Table 5 Layout of CHIP used for analysis of V3 fHbp mutants and their KD values

Mutant number layout

	L1	L2	L3	L4	L5	L6
A1	V3WT	17	11	5	V3WT	Blank
A2	Mut23	18	12	6	Mut4	Blank
A3	24	19	13	7	3	Blank
A4	P46A	20	14	8	2	Blank
A5	P46A	21	15	9	1	Blank
A6	V1WT	22	16	10	V1WT	Blank

Actual mutant layout

	L1	L2	L3	L4	L5	L6
A1	V3WT	Leu117	Ile89	Ser44	V3WT	
A2	Thr128	Ile122	Val91	Pro46	Asp43	
A3	Gln134	Asn123	Asp92	Gln47	Val114	
A4	P46A	Pro125	Ile96	Asn48	His190	
A5	P46A	Asp126	Leu98	Gly49	Lys32	
A6	V1WT	Lys127	Ala99Glu	Gln87	V1WT	

Amount FHbp bound

	L1	L2	L3	L4	L5	L6
A1	0?	1620	3180	1590	3820	
A2	2460	1360	2100	1650	1760	
A3	2140	1830	880	2320	2480	
A4	0?	1770	1970	2180	1270	
A5	0?	1730	1360	2380	1770	
A6	0?	1360	1560	2250	4900	

Run1 Kd

	L1	L2	L3	L4	L5	L6
A1		5.20E-09	3.80E-08	1.00E-08	2.40E-09	
A2	4.00E-09	9.40E-09	5.60E-09	2.60E-11	3.00E-09	
A3	2.60E-09	7.40E-09	4.70E-09	6.00E-08	3.20E-09	
A4		3.20E-09	1.40E-08	3.60E-09	3.40E-09	
A5		6.30E-09	8.60E-08	3.00E-09	3.00E-09	
A6		2.90E-09	7.90E-08	7.10E-10	2.00E-09	

Run2 Kd

	L1	L2	L3	L4	L5	L6
A1		5.20E-09	3.80E-08	9.80E-09	2.30E-09	
A2	4.40E-09	1.10E-08	6.50E-09	3.00E-11	3.40E-09	
A3	2.70E-09	8.60E-09	4.80E-09	5.50E-08	3.30E-09	
A4		3.40E-09	1.50E-08	3.70E-09	3.30E-09	
A5		6.50E-09	8.80E-08	3.00E-09	3.00E-09	
A6		3.00E-09	7.00E-08	7.20E-10	2.10E-09	

Run1 Chi2

	L1	L2	L3	L4	L5	L6
A1		2.7	4.8	2.4	24	
A2	1.10E+01	4.4	5.6	50	2.4	
A3	9	6.8	5.3	4.3	5	
A4		4.6	4.6	4	2.8	
A5		5.3	1.4	2.7	3.7	
A6		5	1.7	3.9	30	

Run2 Chi2

	L1	L2	L3	L4	L5	L6
A1		3.8	5.8	2.6	13	
A2	11.4	4.1	4.5	29	3.9	
A3	11.4	6.6	2.3	5.9	3.5	
A4		4.3	5.9	2.6	2.3	
A5		4.5	1.4	4.4	4.9	
A6		4.2	1.6	3.1	15	

Fold Change

	L1	L2	L3	L4	L5	L6
A1	0.00	2.21	16.17	4.21	1.00	
A2	1.79	4.34	2.57	0.01	1.36	
A3	1.13	3.40	2.02	24.47	1.38	
A4	0.00	1.40	6.17	1.55	1.43	
A5	0.00	2.72	37.02	1.28	1.28	
A6	0.00	1.26	31.70	0.30	0.87	

Average Kd

	L1	L2	L3	L4	L5	L6
A1	0	5.2E-09	3.8E-08	9.9E-09	2.35E-09	
A2	4.2E-09	1.02E-08	6.05E-09	2.8E-11	3.2E-09	
A3	2.65E-09	8E-09	4.75E-09	5.75E-08	3.25E-09	
A4	0	3.3E-09	1.45E-08	3.65E-09	3.35E-09	
A5	0	6.4E-09	8.7E-08	3E-09	3E-09	
A6	0	2.95E-09	7.45E-08	7.15E-10	2.05E-09	

V1 equivalent

	L1	L2	L3	L4	L5	L6
A1	V3WT	Phe174	Ile147	Ser104	V3WT	
A2	Ser185	Ile179	Val149	Arg106	Gln103	
A3	Lys191	Gln180	Asp150	Lys107	Leu171	
A4	P46A	Ser182	Ile154	Asn108	His248	
A5	P46A	Glu183	Leu156	Glu109	Lys92	
A6	V1WT	His184	Glu157	Arg145	V1WT	
