# Supplementary material for: Design and Evaluation of Meningococcal Vaccines through Structure-Based Modification of Host and Pathogen Molecules
Source: PLoS Pathog. 2012 Oct 25;8(10):e1002981. doi: 10.1371/journal.ppat.1002981 (PMC3486911; doi:10.1371/journal.ppat.1002981)
Supplement: Table S6 — Primers used for mutagenesis of V1, V2 and V3 fHbp. (PDF) [file ppat.1002981.s010.pdf]

Supplemental Table 6 Primers used for mutagenesis of V1, V2 and V3 fHbp.
Numbering of the corresponding V1 fHbp residues are shown in the panels for the V2 and \

Mutant Id Number	Forward Primer
Lys92 (Negative Control 1)	gcaccgctcgaccat**gca**gacaaaggtttg
His248 (Negative Control 2)	ggcaaaatcgaa**gct**ttgaaatcgccagaac
Leu171 (Sturctural Control)	caaagccattccgcc**gca**accgcctttcag
Gln103-Ala	cagtctttgacgctggat**gcg**tccgtcagg
Ser104-Ala	ttgacgctggatcag**gcc**gtcaggaaaaac
Arg106-Ala	ctggatcagtccgtc**gcg**aaaaacgagaaac
Lys107-Ala	gatcagtccgtcagg**gca**aacgagaaactg
Asn108-Ala	cagtccgtcaggaaa**gcc**gagaaactgaag
Glu109-Ala	tccgtcaggaaaaac**gcg**aaactgaagctg
Arg145-Ala	cgtttcgactttatc**gcc**caaatcgaagtg
Ile147-Ala	gactttatccgccaa**gcc**gaagtggacggg
Val149-Ala	atccgccaaatcgaa**gcg**gacgggcagctc
Asp150-Ala	cgccaaatcgaagtg**gcc**gggcagctcattac
Ile154-Ala	gtggacgggcagctc**gct**accttggagagtg
Leu156-Ala	gggcagctcattacc**gcg**gagagtggagag
Glu157-Ala	cagctcattaccttg**gcg**agtggagagttc
Phe174-Ala	gccttaaccgcc**gct**cagaccgagcaaatac
Ile179-Ala	tttcagaccgagcaa**gca**caagattcggag
Gln180-Ala	accgagcaaata**gca**gattcggagcattcc
Asp181-Ala	accgagcaaatacaa**gct**tcggagcattcc
Ser182-Ala	gagcaaatacaagat**gcg**gagcattccggg
Glu183-Ala	caaatacaagattcg**gcg**cattccgggaag
His184-Ala	atacaagattcggag**gct**tccgggaagatg
Ser185-Ala	caagattcggagcat**gcc**gggaagatggttg
Lys191-Ala	aagatggttgcg**gca**cgccagttcagaatc
Gln193-Ala	atggttgcgaaacgc**gcg**ttcagaatcggc
Phe194-Ala	gttgcgaaacgccag**gcc**agaatcggcgac
Arg195-Ala	gcgaaacgccagttc**gca**atcggcgacatag
Ile196-Ala	aaacgccagttcaga**gcc**ggcgacatagcg
Ile199-Ala	gaatcggcgac**gca**gcgggcgaacatacatc
His203-Ala	gacatagcgggcgaa**gct**acatcttttgac
Asp262-Ala	gacctggccgccgcc**gct**atcaagccggatg
Lys264-Ala	gccgccgccgatatc**gcg**ccggatggaaaac
Pro265-Ala	gccgccgatatcaag**gcg**gatggaaaacgc
Asp266-Ala	gccgatatcaagccg**gct**ggaaaacgccatg
Lys268-Ala	atcaagccggatgga**gca**cgccatgccgtc

Val272-Ala	ggaaaacgccatgcc**gcc**atcagcggttcc
Ile273-Ala	aaacgccatgccgtc**gcc**agcggttccgtc
Ser274-Ala	catgccgtcatc**gcc**ggttccgtcctttac
Ser286-Ala	gccgagaaaggc**gct**tactccctcggtatc
Ser288-Ala	gagaaaggcagttac**gcc**ctcggtatctttg
Leu289-Ala	aaaggcagttactcc**gcc**ggtatctttggc
Phe292-Ala	tactccctcggtatc**gct**ggcggaaaagcc
Ser302-Ala	gaagttgccggc**gcc**gcggaagtgaaaacc
Lys306-Ala	ggcagcgcggaagtg**gca**accgtaaacggc
Ile311-Ala	aaaaccgtaaacggc**gca**cgccatatcggc
His313-Ala	gtaaacggcatacgc**gct**atcggccttgcc
fHHis402-Tyr	ttggaaaatggatataatcaaaat**tat**ggaagaaagtttgt

/3 proteins

Reverse Primer
caaacctttgtctgcatggtcgagcggtgc
gttctggcgatttcaaagcttcgattttgcc
ctgaaaggcggttgcggcggaatggctttg
cctgacggacgcatccagcgtcaaagactg
gtttttcctgacggcctgatccagcgtcaa
gtttctcgtttttcgcgacggactgatccag
cagtttctcgtttgccctgacggactgatc
cttcagtttctcggctttcctgacggactg
cagcttcagtttcgcgtttttcctgacgga
cacttcgatttgggcgataaagtcgaaacg
cccgtccacttcggcttggcggataaagtc
gagctgcccgtccgcttcgatttggcggat
gtaatgagctgcccggccacttcgatttggcg
cactctccaaggtagcgagctgcccgtccac
ctctccactctccgcggtaatgagctgccc
gaactctccactcgccaaggtaatgagctg
gtatttgctcggtctgagcggcggttaaggc
ctccgaatcttgtgcttgctcggtctgaaa
ggaatgctccgaatctgctatttgctcggt
ggaatgctccgaagcttgtatttgctcggt
cccggaatgctccgcatcttgtatttgctc
cttcccggaatgcgccgaatcttgtatttg
catcttcccggaagcctccgaatcttgtat
caaccatcttcccggcatgctccgaatcttg
gattctgaactggcgtgccgcaaccatctt
gccgattctgaacgcgcgtttcgcaaccat
gtcgccgattctggcctggcgtttcgcaac
ctatgtcgccgattgcgaactggcgtttcgc
cgctatgtcgccggctctgaactggcgttt
gatgtatgttcgcccgctgcgtcgccgattc
gtcaaaagatgtagcttcgcccgctatgtc
catccggcttgatagcggcggcggccaggtc
gttttccatccggcgcgatatcggcggcggc
gcgttttccatccgccttgatatcggcggc
catggcgttttccagccggcttgatatcggc
gacggcatggcgtgctccatccggcttgat

ggaaccgctgatggcggcatggcgttttcc
gacggaaccgctggcgacggcatggcgttt
gtaaaggacggaaccggcgatgacggcatg
gataccgagggagtaagcgcctttctcggc
caaagataccgagggcgtaactgcctttctc
gccaaagataccggcggagtaactgccttt
ggcttttccgccagcgataccgagggagta
ggttttcacttccgcggcgccggcaacttc
gccgtttacggttgccacttccgcgctgcc
gccgatatggcgtgcgccgtttacggtttt
ggcaaggccgatagcgcgtatgccgtttac
ccctgtacaaactttcttccataattttgattatatccatt
