# Supplementary material for: Design and Evaluation of Meningococcal Vaccines through Structure-Based Modification of Host and Pathogen Molecules
Source: PLoS Pathog. 2012 Oct 25;8(10):e1002981. doi: 10.1371/journal.ppat.1002981 (PMC3486911; doi:10.1371/journal.ppat.1002981)
Supplement: Table S7 — Numbering of amino acids in fHbp. (DOCX) [file ppat.1002981.s011.docx]

| **Our numbering** | **Alternate convention** |
| --- | --- |
| Control1 Lys92 | Control1 Lys27 |
| Control2 His248 | Control2 His183 |
| StructControl Leu171 | StructControl Leu106 |
| Gln103 | Gln38 |
| Ser104 | Ser39 |
| Arg106 | Arg41 |
| Lys107 | Lys42 |
| Asn108 | Asn43 |
| Glu109 | Glu44 |
| Arg145 | Arg80 |
| Ile147 | Ile82 |
| Val149 | Val84 |
| Asp150 | Asp85 |
| Ile154 | Ile89 |
| Leu156 | Leu91 |
| Glu157 | Glu92 |
| Phe174 | Phe109 |
| Ile179 | Ile114 |
| Gln180 | Gln115 |
| Asp181 | Asp116 |
| Ser182 | Ser117 |
| Glu183 | Glu118 |
| His184 | His119 |
| Ser185 | Ser120 |
| Lys191 | Lys126 |
| Gln193 | Gln128 |
| Phe194 | Phe129 |
| Arg195 | Arg130 |
| Ile196 | Ile131 |
| Ile199 | Ile134 |
| His203 | His138 |
| Asp262 | Asp197 |
| Lys264 | Lys199 |
| Pro265 | Pro200 |
| Asp266 | Asp201 |
| Gly267 | Gly202 |
| Lys268 | Lys203 |
| Val272 | Val207 |
| Ile273 | Ile208 |
| Ser274 | Ser209 |
| Glu283 | Glu218 |
| Ser286 | Ser221 |
| Ser288 | Ser223 |
| Leu289 | Leu224 |
| Phe292 | Phe227 |
| Ser302 | Ser237 |
| Glu304 | Glu239 |
| Lys306 | Lys241 |
| Ile311 | Ile246 |
| His313 | His248 |
|  |  |

**Supplemental Table 7** showing amino acid numbering of V1 fHbp used here and by others (e.g. Beernik P, et al. (2011) J. Immunol. 186: 3606-14.)
