# Supplementary material for: Design and Evaluation of Meningococcal Vaccines through Structure-Based Modification of Host and Pathogen Molecules
Source: PLoS Pathog. 2012 Oct 25;8(10):e1002981. doi: 10.1371/journal.ppat.1002981 (PMC3486911; doi:10.1371/journal.ppat.1002981)
Supplement: Table S8 — Primers used to modify mfH. (DOC) [file ppat.1002981.s012.doc]

**Supplemental Table 8** Primers used to modify mfH. Altered nucleotides are shown in red. The length of the primers (in base pairs, Bp) and their predicted melting temperatures (Tm, in ^o^C) are shown.

| 5’-3’ sequence (nucleotides in red indicate the introduced change) | Bp | Tm |
| --- | --- | --- |
| ATGGACGTCTGTATCATGAAGAGAGCCGGAGACCCAACTTCC | 42 | 81 |
| TACAGCTATAAGTGTGACGAGCACTTTTCACCACCTTCTGGGTATTCCTG | 52 | 76 |
| CCATGCGTCAGGAAATGTTATTTCCCTTATGTGGAGAATGGAGAC | 45 | 77 |
| GTGGAGAATGGAGACTCTCAAAACTGGGAAAAAGTATATGTGCAG | 45 | 76 |
| GAGAATGGAGACTCTGCATACCACGGAAAAGTATATGTGCAGGGTCAG | 48 | 77 |
| GGAGACTCTGCATACTGGGAAAGAAAATTTGTGCAGGGTCAGTC | 44 | 76 |
