# Supplementary material for: Correction: Design and Evaluation of Meningococcal Vaccines through Structure-Based Modification of Host and Pathogen Molecules
Source: PLoS Pathog. 2013 Jan 17;9(1):10.1371/annotation/3e7e6415-fb12-4a87-89e6-f87d2e800ba8. doi: 10.1371/annotation/3e7e6415-fb12-4a87-89e6-f87d2e800ba8 (PMC3567835; doi:10.1371/annotation/3e7e6415-fb12-4a87-89e6-f87d2e800ba8)
Supplement: Supplementary file 3 [file ppat.3e7e6415-fb12-4a87-89e6-f87d2e800ba8.s003.pdf]

Mutant number layout

	L1	L2	L3	L4	L5	L6
A1	V3WT	17	11	5	V3WT	Blank
A2	Mut23	18	12	6	Mut4	Blank
A3	24	19	13	7	3	Blank
A4	P46A	20	14	8	2	Blank
A5	P46A	21	15	9	1	Blank
A6	V1WT	22	16	10	V1WT	Blank

Actual mutant layout

	L1	L2	L3	L4	L5	L6
A1	V3WT	Leu117	Ile89	Ser44	V3WT	
A2	Thr128	Ile122	Val91	Pro46	Asp43	
A3	Gln134	Asn123	Asp92	Gln47	Val114	
A4	P46A	Pro125	Ile96	Asn48	His190	
A5	P46A	Asp126	Leu98	Gly49	Lys32	
A6	V1WT	Lys127	Ala99Glu	Gln87	V1WT	

V1 equivalent

	L1	L2	L3	L4	L5	L6
A1	V3WT	Phe174	Ile147	Ser104	V3WT	
A2	Ser185	Ile179	Val149	Arg106	Gln103	
A3	Lys191	Gln180	Asp150	Lys107	Leu171	
A4	P46A	Ser182	Ile154	Asn108	His248	
A5	P46A	Glu183	Leu156	Glu109	Lys92	
A6	V1WT	His184	Glu157	Arg145	V1WT	

Amount FHbp bound

	L1	L2	L3	L4	L5	L6
A1	0?	1620	3180	1590	3820	
A2	2460	1360	2100	1650	1760	
A3	2140	1830	880	2320	2480	
A4	0?	1770	1970	2180	1270	
A5	0?	1730	1360	2380	1770	
A6	0?	1360	1560	2250	4900	

Run1 Kd

	L1	L2	L3	L4	L5	L6
A1		5.20E-09	3.80E-08	1.00E-08	2.40E-09	
A2	4.00E-09	9.40E-09	5.60E-09	2.60E-11	3.00E-09	
A3	2.60E-09	7.40E-09	4.70E-09	6.00E-08	3.20E-09	
A4		3.20E-09	1.40E-08	3.60E-09	3.40E-09	
A5		6.30E-09	8.60E-08	3.00E-09	3.00E-09	
A6		2.90E-09	7.90E-08	7.10E-10	2.00E-09	

Run1 Chi2

	L1	L2	L3	L4	L5	L6
A1		2.7	4.8	2.4	24	
A2	1.10E+01	4.4	5.6	50	2.4	
A3	9	6.8	5.3	4.3	5	
A4		4.6	4.6	4	2.8	
A5		5.3	1.4	2.7	3.7	
A6		5	1.7	3.9	30	

Run2 Kd

	L1	L2	L3	L4	L5	L6
A1		5.20E-09	3.80E-08	9.80E-09	2.30E-09	
A2	4.40E-09	1.10E-08	6.50E-09	3.00E-11	3.40E-09	
A3	2.70E-09	8.60E-09	4.80E-09	5.50E-08	3.30E-09	
A4		3.40E-09	1.50E-08	3.70E-09	3.30E-09	
A5		6.50E-09	8.80E-08	3.00E-09	3.00E-09	
A6		3.00E-09	7.00E-08	7.20E-10	2.10E-09	

Run2 Chi2

	L1	L2	L3	L4	L5	L6
A1		3.8	5.8	2.6	13	
A2	11.4	4.1	4.5	29	3.9	
A3	11.4	6.6	2.3	5.9	3.5	
A4		4.3	5.9	2.6	2.3	
A5		4.5	1.4	4.4	4.9	
A6		4.2	1.6	3.1	15	

Average Kd

	L1	L2	L3	L4	L5	L6
A1	0	5.2E-09	3.8E-08	9.9E-09	2.35E-09	
A2	4.2E-09	1.02E-08	6.05E-09	2.8E-11	3.2E-09	
A3	2.65E-09	8E-09	4.75E-09	5.75E-08	3.25E-09	
A4	0	3.3E-09	1.45E-08	3.65E-09	3.35E-09	
A5	0	6.4E-09	8.7E-08	3E-09	3E-09	
A6	0	2.95E-09	7.45E-08	7.15E-10	2.05E-09	

Fold Change

	L1	L2	L3	L4	L5	L6
A1	0.00	2.21	16.17	4.21	1.00	
A2	1.79	4.34	2.57	0.01	1.36	
A3	1.13	3.40	2.02	24.47	1.38	
A4	0.00	1.40	6.17	1.55	1.43	
A5	0.00	2.72	37.02	1.28	1.28	
A6	0.00	1.26	31.70	0.30	0.87	

Mutant number layout

	L1	L2	L3	L4	L5	L6
A1	WTV1	41	35	29	WTV3	Blank
A2	Mut47	42	36	30	25	Blank
A3	48	43	37	31	26	Blank
A4	P46A	44	38	32	27	Blank
A5	P46A	45	39	33	28	Blank
A6	WTV3	46	40	34	WTV1	Blank

Actual mutant layout

	L1	L2	L3	L4	L5	L6
A1	WTV1	His288	Lys268	Leu199	WTV3	
A2	Val311	Leu289	Val272	His203	Ser193	
A3	Glu313	Phe292	Ile273	Glu262	Phe194	
A4	P46A	Ser302	Leu274	Lys264	Leu195	
A5	P46A	Thr304	Glu283	Ala265Pro	Val196	
A6	WTV3	Lys306	Thr286	Glu267	WTV1	

V1 equivalent

	L1	L2	L3	L4	L5	L6
A1	WTV1	Ser288	Lys268	Ile199	WTV3	
A2	Ile311	Leu289	Val272	His203	25.Gln193	
A3	Glu255	Phe292	Ile273	Asp262	26.Phe194	
A4	P46A	Ser302	Ser274	Lys264	27.Arg195	
A5	P46A	Glu304	Glu283	Pro265	28.Ile196	
A6	WTV3	Lys306	Ser286	Gly267	WTV1	

Amount FHbp bound

	L1	L2	L3	L4	L5	L6
A1	4040	2090	2120	1820	840	
A2	930	1870	1580	1840	1000	
A3	1600	1250	1570	1450	770	
A4	2260	1290	2140	1880	780	
A5	2530	810	2420	1790	1070	
A6	705	930	1370	1440	3430	

Run1 Kd

	L1	L2	L3	L4	L5	L6
A1	1.80E-09	6.40E-09	1.70E-09	3.20E-08	2.80E-09	
A2	1.70E-08	7.80E-09	2.20E-08	2.00E-09	3.70E-09	
A3	NBD	1.70E-09	2.70E-10	6.00E-09	4.00E-09	
A4	5.70E-11	5.90E-09	1.90E-08	1.90E-08	NBD	
A5	5.00E-11	1.90E-08	2.00E-08	3.20E-09	1.40E-08	
A6	2.80E-09	9.30E-09	5.60E-04	1.10E-09	1.70E-09	

Run1 Chi2

	L1	L2	L3	L4	L5	L6
A1	61?	13.6	18.4	3.9	4.9	
A2	1.3	10	3.3	14.8	3.6	
A3	NBD	4.2	12.8	10	1.2	
A4	17	5.7	6	4.1	NBD	
A5	20	1.8	5.3	6.8	3.1	
A6	4.3	3.1	1.2	4.2	24	

Run2 Kd

	L1	L2	L3	L4	L5	L6
A1	1.90E-09	7.00E-09	1.90E-09	3.90E-08	2.90E-09	
A2	2.00E-08	1.00E-08	2.60E-08	2.10E-09	3.80E-09	
A3	NBD	1.80E-09	3.00E-10	6.00E-09	4.00E-09	
A4	6.70E-11	6.10E-09	2.10E-08	1.90E-08	NBD	
A5	5.60E-11	1.80E-09	1.90E-08	3.30E-09	1.30E-08	
A6	2.80E-09	9.50E-09	1.30E-07	1.20E-09	1.80E-09	

Run2 Chi2

	L1	L2	L3	L4	L5	L6
A1	23	7.9	5.6	4.7	6.3	
A2	1	3.3	1.7	4.6	2.6	
A3	NBD	2.3	4.8	4	1.1	
A4	5.3	3.4	3.3	3.2	NBD	
A5	7.1	1.7	5	7	2.9	
A6	3.1	2.5	1.4	3.8	17	

Average Kd

	L1	L2	L3	L4	L5	L6
A1	1.85E-09	6.7E-09	1.8E-09	3.55E-08	2.85E-09	
A2	1.85E-08	8.9E-09	2.4E-08	2.05E-09	3.75E-09	
A3	NBD	1.75E-09	2.85E-10	6E-09	4E-09	
A4	6.2E-11	6E-09	0.00000002	1.9E-08	NBD	
A5	5.3E-11	1.04E-08	1.95E-08	3.25E-09	1.35E-08	
A6	2.8E-09	9.4E-09	1.30E-07	1.15E-09	1.75E-09	

Fold Change

	L1	L2	L3	L4	L5	L6
A1	0.66	2.39	0.64	12.68	1.02	
A2	6.61	3.18	8.57	0.73	1.34	
A3	NBD	0.63	0.10	2.14	1.43	
A4	0.02	2.14	7.14	6.79	NBD	
A5	0.02	3.71	6.96	1.16	4.82	
A6	1.00	3.36	46.43	0.41	0.63	
